# Revisiting the Role of Charge Transfer in the Emission Properties of Carborane–Fluorophore Systems: A TDDFT Investigation

**DOI:** 10.1021/acs.jpca.2c02435

**Published:** 2022-06-05

**Authors:** Duygu Tahaoğlu, Hakan Usta, Fahri Alkan

**Affiliations:** Department of Nanotechnology Engineering, Abdullah Gül University, Kayseri 38080, Turkey

## Abstract

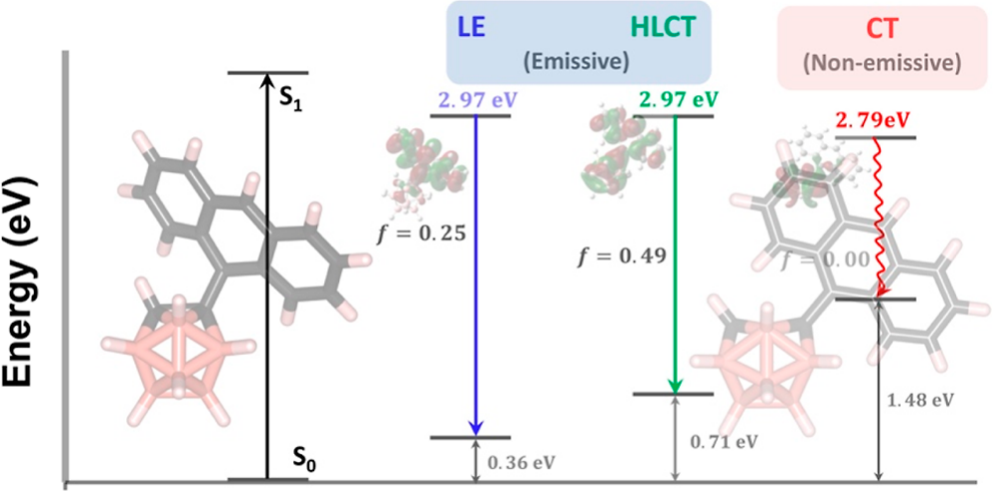

In this study, we performed a detailed investigation of the S_1_ potential energy surface (PES) of *o*-carborane–anthracene (*o*-CB–*Ant*) with respect to the C–C bond length on *o*-CB and the dihedral angle between *o*-CB and *Ant* moieties. The effects of different substituents (F, Cl, CN, and OH) on carbon- or boron-substituted *o*-CB, along with a π-extended acene-based fluorophore, pentacene, on the nature and energetics of S_1_ → S_0_ transitions are evaluated. Our results show the presence of a non-emissive S_1_ state with an almost pure charge transfer (CT) character for all systems as a result of significant C–C bond elongation (C–C = 2.50–2.56 Å) on *o*-CB. In the case of unsubstituted *o*-CB–*Ant*, the adiabatic energy of this CT state corresponds to the global minimum on the S_1_ PES, which suggests that the CT state could be involved in emission quenching. Despite large deformations on the *o*-CB geometry, predicted energy barriers are quite reasonable (0.3–0.4 eV), and the C–C bond elongation can even occur without a noticeable energy penalty for certain conformations. With substitution, it is shown that the dark CT state becomes even more energetically favorable when the substituent shows −M effects (e.g., −CN), whereas substituents showing +M effects (e.g., −OH) can result in an energy increase for the CT state, especially for partially stretched C–C bond lengths. It is also shown that the relative energy of the CT state on the PES depends strongly on the LUMO level of the fluorophore as this state is found to be energetically less favorable compared to other conformations when anthracene is replaced with π-extended pentacene. To our knowledge, this study shows a unique example of a detailed theoretical analysis on the PES of the S_1_ state in *o*-CB–*fluorophore* systems with respect to substituents or fluorophore energy levels. Our findings could guide future experimental work in emissive *o*-CB–*fluorophore* systems and their sensing/optoelectronic applications.

## 1 Introduction

Fluorescent π-conjugated molecules have attracted tremendous interest in the last few decades as functional materials for both fundamental photophysical studies and in new-generation light-emitting and sensing applications ranging from life sciences to optoelectronics.^1−5^ Especially, the ability to engineer fluorescent π-frameworks with electron-accepting and -donating substituents or (hetero)aromatic building blocks has enabled unprecedented diversity and fine-tuning ability in chemical structures and optoelectronic/sensing characteristics.^6−8^ To this end, one of the unconventional approaches include carboranes, which are non-classically bonded clusters of boron, carbon, and hydrogen atoms. Among carboranes, icosahedral *closo*-carborane (C_2_B_10_H_12_) stands out as a highly stable neutral framework as described by Wade-Mingos rules.^9−11^ In icosahedral *closo*-carboranes, three isomeric forms showing different polarities^12^ and electronic acceptor capabilities (para < meta ≪ ortho)^13,14^ are plausible [1,2-C_2_B_10_H_12_ (*o*-carborane), 1,7-C_2_B_10_H_12_ (*m*-carborane), and 1,12-C_2_B_10_H_12_ (*p*-carborane)]. Among them, *o*-carborane containing two adjacent carbon atoms is the most studied cluster based on its facile reaction with varied π-systems to yield chemically and thermally stable molecules with potential applications in catalysis, electronics, energy storage, and medicine.^15−23^

More recently, *o*-carborane has drawn attention as a building block to modify the chemical structures and to tune the fluorescence properties of π-conjugated fluorophores.^21,24−40^*o*-carborane shows distinct electron delocalization via three-center two-electron bonds, which, along with the presence of boron atoms, gives strong electron-withdrawing ability to this cluster framework.^28^ Therefore, when *o*-carborane is tethered to a relatively π-electron-rich fluorophore at one of its carbon positions via a C–C single bond, donor–acceptor type electronic structures with tunable intramolecular charge transfer (ICT) characteristics could be realized.^40,41^ While the *o*-carborane–fluorophore adducts typically exhibit low photoluminescence quantum yields in varied solutions, a unique enhanced emission behavior can be observed in the solid-state (i.e., aggregation-induced emission).^33,38,42−44^ This has been discussed in the previous literature that restricting the undesired vibrations of *o*-carborane’s “C–C” bond in the aggregate state leads to enhanced photoluminescence quantum yields. In addition, it has been shown that the enhancement in emission could also be achieved in the solution phase via structural modifications on *o*-carborane to limit *o*-carborane’s “C–C” bond elongation, which plays a critical role in emission quenching mechanisms.^38,43,45^ It is also noteworthy that the dihedral angle between the *o*-carborane’s “C–C” bond and the fluorophore is another key parameter affecting the emission properties and tuning dual emission characteristics.^42,46−50^ A similar interplay between emission properties and cluster–fluorophore orientation is also seen for boron hydride subclusters and pyridine ligands.^51^

Among *o*-carborane–fluorophore adducts studied to date, the *o*-carborane–anthracene (*o*-CB–*Ant*) molecule and its derivatives have attracted significant attention for both theoretical and synthetic studies since the seminal works by Chujo and co-workers.^24,25^ In solution, *o*-CB–*Ant* shows dual emissions with low quantum yields, where the high-energy S_1_ → S_0_ transition has a local excited (LE)-state character on anthracene, whereas the low-energy transition shows twisted ICT (TICT). The TICT state mainly arises from the strong interaction between *o*-carborane’s “C–C” bond and anthracene’s π-conjugated system, along with the perpendicular rearrangement of the C–C bond with respect to the plane of anthracene.^52^ This TICT emission can be maintained even in aggregated or crystal states due to the presence of sufficient space for rotations as a result of *o*-CB’s compact spherical structure.^24,42,52^ On the other hand, the photophysical properties of the *o*-CB–*Ant* system can be manipulated via functionalization or substitution of the adjacent C atoms on *o*-carborane. For example, while only a high-energy TICT state with a low quantum yield is observed in solution with methyl or phenyl groups, a substitution with bulky groups (e.g., trimethylsilyl) leads to a significant increase in the quantum yield.^22^ More recently, Duan et al. have investigated the *o*-CB–*Ant* derivatives using quantum mechanical and molecular dynamics simulations, based on which the elongation of C–C bond is suggested to lead to bathochromically shifted emissions with an increased CT character for the S_1_ → S_0_ transition.^52^

In light of these recent studies, it has been mostly suggested that *o*-carborane’s “C–C” bond elongation and the relative orientations of fluorophore versus *o*-carborane moieties govern the electronic interaction and, hence, the photophysical properties of *o*-CB–*fluorophore* systems. However, a complete theoretical understanding is still lacking in the literature for the interplay of excited-state nature/energetics and potential energy surfaces (PESs) with regard to emissive transitions and quenching mechanisms. Thus, it is still of great importance to pursue theoretical investigations on novel *o*-CB–*fluorophore* systems with the motivation of revealing the key effects of structural modifications and providing a better understanding of the photophysical properties. To this end, we herein perform a detailed investigation for the PESs of the S_1_ state for *o*-CB–*Ant*. Furthermore, we investigate the role of different substituents (F, Cl, CN, and OH) in the *o*-CB cluster along with a π-extended acene-based fluorophore, pentacene, (*o*-CB–*Pnt*) on the energetics and the nature of S_1_ → S_0_ transitions for different conformations. Our results indicate the presence of a non-emissive CT state for *o*-CB–*Ant* as a result of significant C–C bond elongation in *o*-CB, which is suggested to play an important role for emission quenching as this state also corresponds to the lowest-energy excited state on the S_1_ PES in our investigation. Our results also show that the relative energy of this non-emissive CT state and energy barriers on the S_1_ PES can be modulated with respect to substituents or fluorophore energy levels, which can guide future experimental work in terms of emission tuning and enhancement for *o*-CB–*fluorophore* systems.

## 2 Computational Methods

All computations were performed using the Gaussian09^53^ program package. The ground-state geometries of the investigated *o*-CB–*Ant*, substituted *o*-CB–*Ant*, and *o*-CB–*Pnt* systems were optimized at the M06-2X/6-31G* level of theory. No imaginary frequencies were found for the optimized molecules. An integral equation formalism variant of the polarizable continuum model^54−58^ was employed using THF as the solvent for both ground-state and excited-state computations. The MO diagrams were visualized by using GaussView,^59^ and orbital contributions were generated on GaussSum^60^ packages. Excited-state computations were performed using TDDFT formalism as implemented in Gaussian09. The Multiwfn program^61^ was employed to calculate the charge separation parameter (Δ*r*) for the excited states, degree of overlap (∧) indexes of hole and electron wave functions, and the heat maps of transition density matrices (TDMs). Energy barriers for the excited-state PESs were examined both by alteration of the dihedral angle (φ) with fixed C–C bond length and by C–C bond elongation with fixed φ.

**Figure 1:**
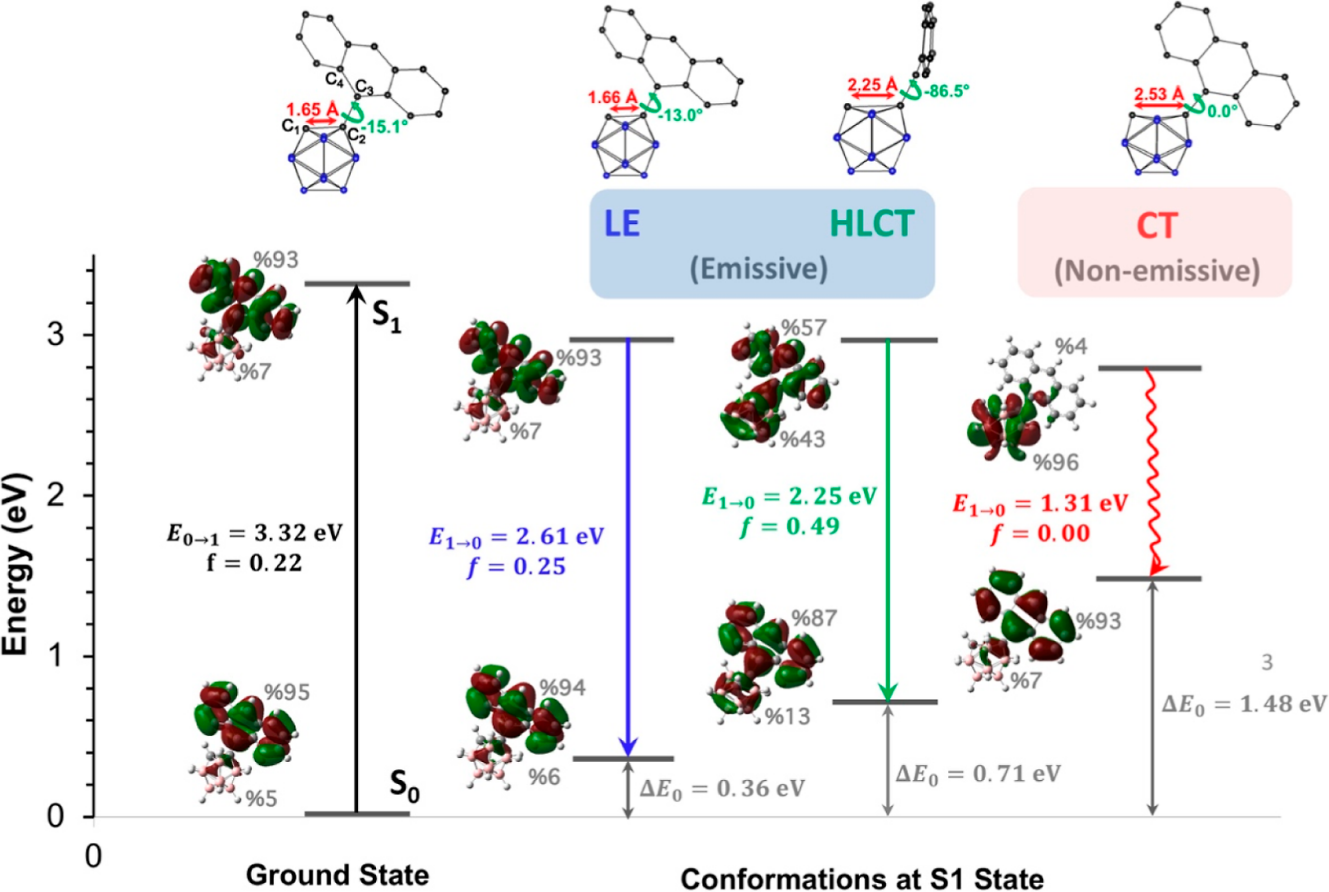
Illustration of the absorption and potential emission paths along with the corresponding geometries of the o-CB–*Ant* system. Transition energies (***E***_0→1_/***E***_1→0_) and the oscillator strengths (***f***) are shown for each process. Adiabatic S_1_ state energies (***E***_**S**1_) were calculated by the addition of ***E***_0→1_/***E***_1→0_ for the absorption and emissive/non-emissive processes to the ground-state energies (Δ***E***_0_) at corresponding geometric conformations. These three conformations correspond to the minimum energy points on the excited-state (S_1_) PES. % contribution of anthracene and *o*-CB-based orbitals to HOMO and LUMO for each transition are shown on the orbital pictures.

As shown in a previous work,^24^ there are two main degrees of freedom for the geometry of the *o*-CB–*Ant* system: the dihedral angle [φ *for* (C_1_–C_2_)–(C_3_–C_4_)] between the carborane and anthracene moieties and the C_1_–C_2_ bond length in the *o*-carborane cluster as shown with green and red arrows, respectively, along with the numbering of C atoms shown in Figure S1. Two conformations of this system for the S_1_ state have been explored previously.^24^ These two conformations are shown in Figure 1, and they correspond to the LE and the hybridized local and charge-transfer (HLCT) excited states, respectively. We note that the second conformation has often been referred to as the TICT state in earlier studies; however, our investigation reveals that this excited state shows a good mixture of both LE and CT characters and, thus, it is referred to as the HLCT excited state in our work. In addition, in our analysis of the S_1_ state, a new conformation corresponding to a pure CT character (the rightmost conformation in Figure 1) is revealed (vide infra) for the first time in the literature as a result of further elongation of the C_1_–C_2_ bond. We also note that Ji et al.^62^ have shown the existence of a similar CT state (C_1_–C_2_ = 2.62 Å) for a 1-(pyren-2-yl)-*o*-carborane system in a very recent study. In this work, this state is named as the S_1_-M (mixed) state, while the twisted confirmation for the 1-(pyren-2-yl)-*o*-carborane system is called S_1_-CT.

Different functionals [BP86^63,64^ (GGA), B3LYP^65^ (hybrid), CAM-B3LYP^66^ (range-separated), and M06-2X^67^ (meta-hybrid)] were employed for the TDDFT optimization of three main excited-state conformations for benchmarking purposes. We note that B3LYP has been widely preferred for the excited-state investigation of *o*-CB–*Ant* as well as other carborane–fluorophore systems.^24,28−33,40,42,47,68−70^ In general, this functional shows good agreement for the absorption and emission energies of these systems. However, one should also note that it can sometimes be problematic for pure CT states as it is shown to underestimate the excited-state energies for such transitions.^71−75^ In Tables S1–S3 and Figure S3, we compare the results of our benchmark calculations. As shown in the table, BP86 significantly underestimates the experimental results as expected. In comparison, B3LYP shows some improvements toward experimental values as the predicted energies of the vertical S_0_ → S_1_ and S_1_ → S_0_ transitions of LE and HLCT states are 0.19–0.33 eV higher than those with BP86. Overall, the performances of M06-2X and CAM-B3LYP functionals are better when compared with the B3LYP functional, especially for the S_1_ → S_0_ transitions of the LE state. However, the largest deviation (∼0.6 eV) between B3LYP and M06-2X/CAM-B3LYP is seen for the S_1_ → S_0_ transition energy of the CT state as expected. BP86 and B3LYP functionals are also found to overestimate the degree of CT, particularly for HLCT and CT states (Figure S3). Therefore, we concluded that both BP86 and B3LYP functionals are not suitable for the investigation of the excited-state PES due to the involvement of a pure CT character for certain geometry conformations. Meanwhile, the difference between the predicted transition energies via M06-2X and CAM-B3LYP is within 0.1 eV for all states. There is also a good agreement for the predicted Δ*r* and ∧ parameters for these functionals. We note that the M06-2X functional has been shown to perform better for cluster systems compared to other functionals in terms of bonding and structure predictions.^76,77^ In addition, this functional generally showed more stable excited-state geometry optimizations when constraints were involved in our test calculations as compared to CAM-B3LYP. Therefore, we used the M06-2X/6-31G* level of theory for the rest of our investigation.

## 3 Results and Discussion

### 3.1 *o*-CB–*Ant* System

In Figure 1, we show the energetics, excited-state geometries, along with the excited-state characteristics for the vertical S_0_ → S_1_ transition (absorption) and the possible S_1_ → S_0_ pathways (emission) for the *o*-CB–*Ant* system. In the case of the vertical S_0_ → S_1_ transition, and the S_1_ → S_0_ transition in the LE conformation, the excited states mainly originate from the π–π* transition localized on the anthracene π-system, and the calculated oscillator strengths are similar (*f* = 0.22 vs. 0.25). The transition energies for vertical S_0_ → S_1_ (E_0→1_) and S_1_→ S_0_ (E_1→0_) in the LE state are calculated to be 3.32 and 2.61 eV, respectively. On the other hand, as a result of the C_1_–C_2_ bond elongation (1.66 Å → 2.25 Å) and the alteration of the dihedral angle (−13.0° → −86.5°) as shown in Figure 1, another possible S_1_ → S_0_ pathway is predicted with an HLCT character, a reduced transition energy (E_1→0_ = 2.25 eV), and an increased oscillator strength (*f* = 0.49). At this point, two important parameters play major roles in the electronic structures and the resulting excited-state energetics/characteristics. First, the energy of the LUMO level for the *o*-carborane moiety can be altered significantly with the C_1_–C_2_ bond length as this level shows a large antibonding character between C_1_ and C_2_. The comparison of the frontier orbital energy levels for varying C_1_–C_2_ bond lengths is shown in Figure S4. Second, the degree of orbital mixing between anthracene and *o*-carborane LUMO levels can be altered with φ, where, unlike traditional push–pull systems, φ = −90° corresponds to a maximum coupling between these orbitals, while φ = 0° corresponds to a minimum coupling. As a result, elongated C_1_–C_2_ bond length (2.25 Å) and the large φ (−87°) induce a strong orbital mixing between *o*-carborane and anthracene originated levels for the electron wavefunction of the HLCT state as shown in Figure 1. It should be noted that our findings are in good agreement with the previous findings by Chujo and co-workers^24^ as the emission (S_1_ → S_0_) is experimentally shown to be possible from both LE and HLCT states.

As shown in Figure S4, it is possible for the *o*-carborane LUMO level to be considerably lower in energy than that of anthracene with elongation of the C_1_–C_2_ bond. This is indeed the case for the formation of the pure CT state of *o*-CB–*Ant* where the C_1_–C_2_ bond length increases to 2.53 Å. In addition, since φ becomes 0°, the orbital mixing between *o*-carborane- and anthracene-based levels is restricted by symmetry. As a result, the corresponding S_1_ → S_0_ transition in this conformation is of a strong CT character with a vanishing oscillator strength, where holes and electrons are spatially separated and localized on the anthracene and the *o*-carborane moieties, respectively. The energy of the S_0_ state for the CT state geometry is calculated to be much higher (Δ*E*_0_ = 1.48 eV) than that of the ground-state geometry. In comparison, the same energy differences for HLCT and LE states are 0.71 and 0.36 eV, respectively. Despite this large deviation from the minimum S_0_, the adiabatic energy of the excited state (*E*_S1_) for the CT state is 2.79 eV, which is not only lower than those of both LE and HLCT states but also the predicted minimum energy on the S_1_ PES in our investigation. This is, of course, related to the fact that *E*_1→0_ becomes significantly smaller (1.31 eV), indicating an energetically close point between S_1_ and S_0_ surfaces.

**Figure 2:**
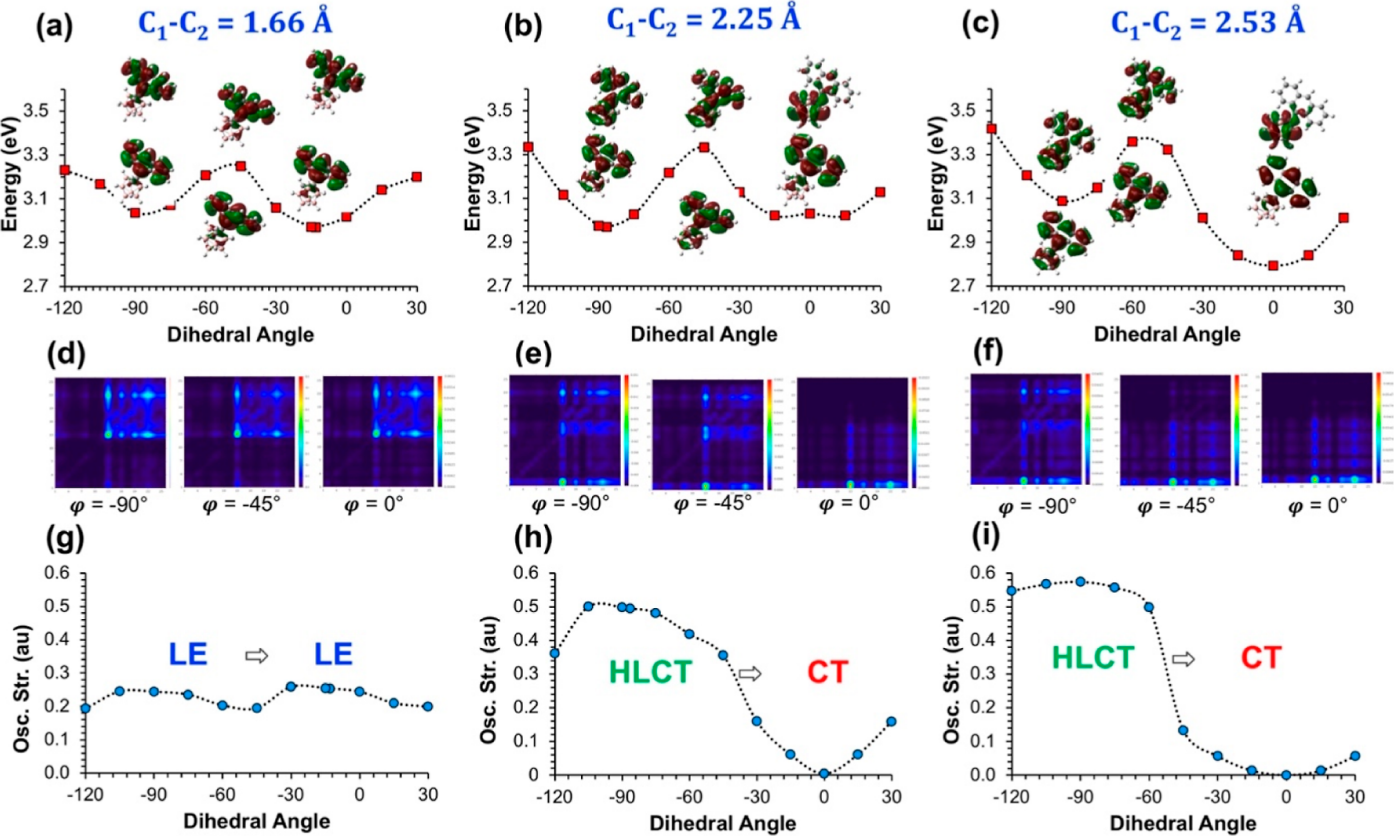
(a–c) PESs for the adiabatic excited-state energies (*E*_S1_) with respect to φ at fixed C_1_–C_2_ bond lengths, (d-f) heat maps of TDM graphs for selective S_1_ states calculated with M06-2X/6-31g* (Δ*r* and Λ indexes are given in Table S4), and (g–i) calculated oscillator strengths for the corresponding S_1_ → S_0_ transitions. Frontier orbital pictures are also given for some specific conformations on PES diagrams to show the excited-state character change. Atom numbering for the TDM plots is shown in Figure S2.

The excited-state optimization of *o*-CB–*Ant* reveals three critical C_1_–C_2_ bond lengths. While the CT state exhibits the minimum energy for S_1_, it is also important to understand the energy barriers on PESs and the excited-state characteristics of the S_1_ state for different conformations. In Figure 2, we show the PESs for the S_1_ state with respect to φ for fixed C_1_–C_2_ bond lengths (Figure 2a–c), calculated TDMs for selective S_1_ states on this surface (Figure 2d–f), and oscillator strengths for the corresponding S_1_ → S_0_ transitions (Figure 2g–i). When the C_1_–C_2_ bond length is 1.66 Å, the energy of S_1_ is minimum for φ = −13° (LE state), while there is a local minimum at −90° with a very similar energy. It is seen that there is a rotational barrier (∼0.3 eV) with changing φ where the maximum energy is calculated for the conformation where φ = −45°. For this surface, S_1_ → S_0_ transitions mainly originate from the π–π* transitions of anthracene for all conformations, regardless of φ. As a result, the calculated oscillator strengths along with ∧ and Δr parameters (Table S4) are quite similar and show strong LE characteristics for all φ values.

**Figure 3:**
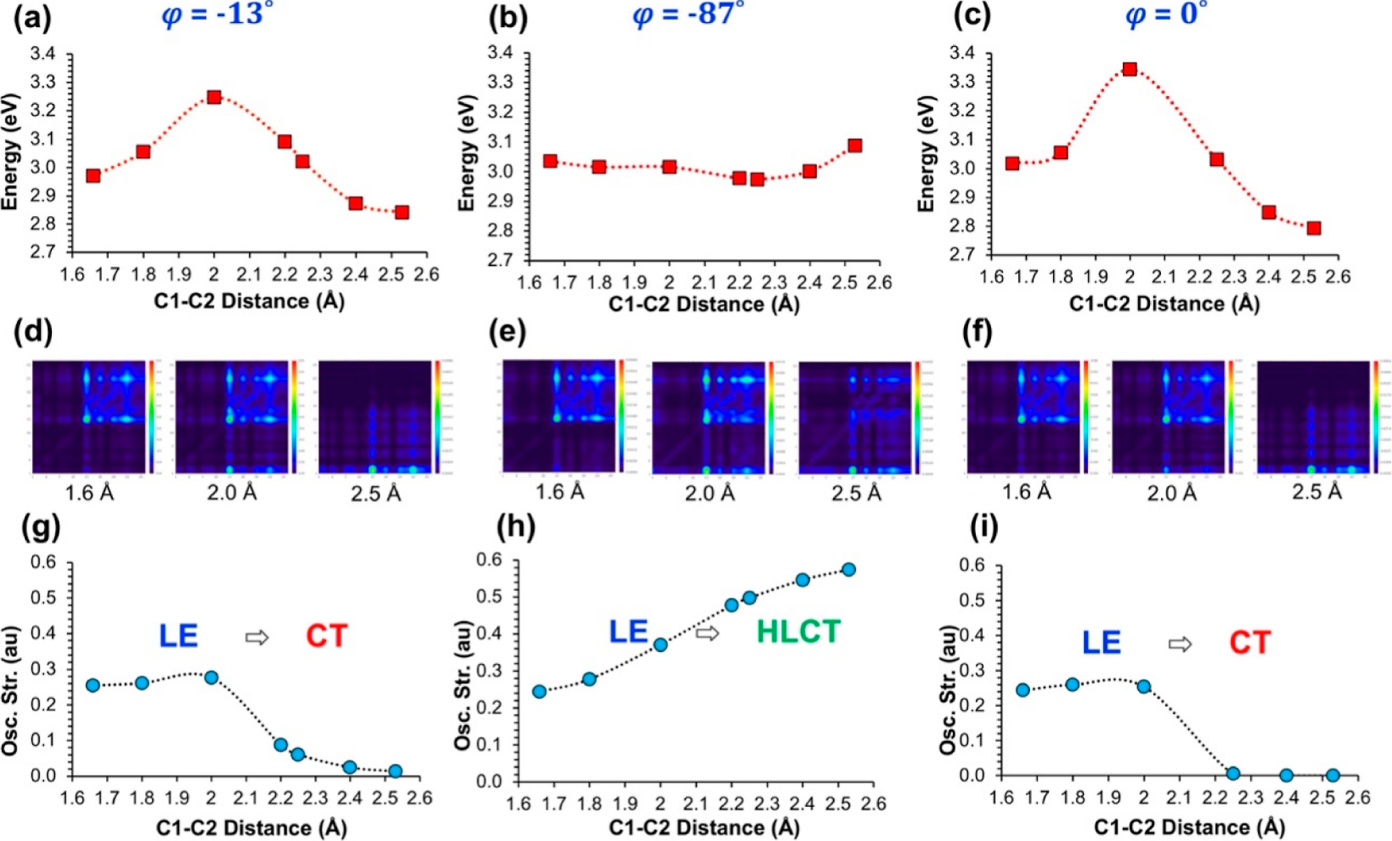
(a–c) PESs for the adiabatic excited-state energies (*E*_S1_) with respect to C_1_–C_2_ bond lengths at fixed φ, (d–f) TDMs for selective S_1_ states (Δ*r* and Λ indexes are given in Table S5), and (g–i) calculated oscillator strengths for the corresponding S_1_ → S_0_ transitions. Atom numbering for the TDM plots is given in Figure S2.

When the C_1_–C_2_ bond partially stretches to 2.25 Å, the minimum *E*_S1_ (2.97 eV) corresponds to the twisted conformation with φ = −87°. Similar to the case where the C_1_–C_2_ bond length is 1.66 Å, there is a rotational barrier with a slightly increased energy of ∼0.4 eV, and the conformation with φ = −45° corresponds to the local maximum (energy = 3.33 eV). In this case, however, the nature of the S_1_ → S_0_ transitions shows a significant alteration with φ, as indicated in the oscillator strengths trend (Figure 3h), along with calculated TDMs (Figure 3e) and ∧ or Δ*r* parameters (Table S4). When φ is close to −90°, the S_1_ → S_0_ transition exhibits an HLCT character where the hole wave function is mainly localized on anthracene, while the electron wave function extends to both *o*-carborane and anthracene moieties. As a result, the Δr index shows a significant increase for this transition compared to the LE case, while ∧ shows a slight decrease. In addition, the CT character becomes more dominant as φ changes from −90 to 0°. At φ = 0°, the total energy of S_1_ (3.02 eV) is comparable to that with φ = −87°; however, the calculated oscillator strength for the S_1_ → S_0_ transition vanishes as a result of the strong CT character at this point. For this case, TDM and MO analyses reveal that the electron wave function is localized on the *o*-carborane cluster, while the hole wave function is localized on the anthracene moiety.

For conformations where the C_1_–C_2_ bond is fully elongated to 2.53 Å, the origin of the S_1_ → S_0_ transition with respect to φ shows a similar trend to the case where the C_1_–C_2_ bond is 2.25 Å as illustrated by calculated oscillator strengths and TDMs in Figure 2. As to the other PESs, there is a rotational barrier with a local maximum located at between −45 and −60°. In general, the CT character for the S_1_ → S_0_ transitions on this surface is more pronounced compared to the other cases due to increasing contribution from the *o*-carborane-based orbitals to the electron wave function. Another important point is that the minimum point (2.79 eV) occurs at φ = 0° (CT state), with a significantly lower energy than the local minima at φ = −90° (3.09 eV) and the other minima (LE and HLCT states) in previous PESs (2.97 eV). As mentioned earlier, this conformation corresponds to the global minimum for the calculated *E*_S1_ in our investigation.

Similar to our analysis with φ, Figure 3a–c shows the calculated PESs of the S_1_ state for the C_1_–C_2_ bond elongation for fixed φ values at −13, −87, and 0°, respectively. In addition, the TDMs for selected points (Figure 3d–f) and the calculated oscillator strengths (Figure 3g–i) are given to investigate the origin of the corresponding S_1_ → S_0_ transitions along these surfaces. It should be noted that the PESs and the oscillator strengths at φ = −13° and φ = 0° show a similar trend, which originates from the fact that orbital mixing between *o*-carborane- and anthracene-based levels is similarly restricted for such small φ values. In both cases, the PESs show an energy barrier of ∼0.3 eV with bond elongations at a maximum point of ∼2.0 Å. Interestingly, this bond length also corresponds to the crossover point for S_1_ → S_0_ transitions from LE to CT character as indicated from the calculated oscillator strengths. This is, of course, related to the relative energies of the *o*-carborane-based and anthracene-based frontier orbitals for the unoccupied levels (Figure S4) in the electronic structure. When φ is −87°, however, the calculated PES becomes quite flat, indicating that C_1_–C_2_ bond elongation can occur on this surface without an energy penalty. Another important point is that the S_1_ → S_0_ transition becomes increasingly HLCT in character with C_1_–C_2_ elongation as shown by the increase of the corresponding oscillator strengths (Figure 3h) and TDMs (Figure 3e).

Previous experimental and theoretical works on *o*-CB–*Ant* and its derivatives have shown that these systems can facilitate dual emission in solution and solid state through LE and HLCT (or TICT) states as a result of intramolecular rotation upon photoexcitation. In addition, these systems generally exhibit low quantum yields in solution, which is often associated with the vibrational motion of the C_1_–C_2_ bond. More recently, Ochi et al.^45,78^ have demonstrated that for carbon–boron fused carboranes, C_1_–C_2_ bond elongation can cause emission quenching without an intramolecular rotation. In our investigation for the S_1_ state of *o*-CB–*Ant*, it is revealed that C_1_–C_2_ bond elongation to 2.53 Å leads to a non-emissive S_1_ → S_0_ transition with a strong CT character and a vanishing oscillator strength, which also corresponds to the lowest-energy point on the S_1_ PES. While this conformation exhibits a highly energetic S_0_ point (1.48 eV as shown in Figure 1), the calculated energy barriers on the S_1_ surfaces are quite reasonable (0.3–0.4 eV), suggesting that the molecule can reach this geometry via electronic-vibronic couplings upon photoexcitation. In a very recent study, the existence of a similar CT state has also been confirmed for the 1-(pyren-2-yl)-*o*-carborane system, where the C_1_–C_2_ bond length becomes 2.62 Å.^62^ In both cases, the energy gap between the S_0_ and the S_1_ surfaces becomes quite small around this point, which can further increase the nonradiative decay rate according to the energy gap law. These findings along with previous experimental evidence suggest that the CT state resulting from a fully elongated C_1_–C_2_ bond could be an important pathway on the fluorescence quenching of *o*-CB–*Ant* and its derivatives. It should also be noted that fluorescence quenching through the CT state may not be the sole mechanism for *o*-CB–fluorophore systems where a parallel conformation between the C_1_–C_2_ bond and fluorophores (φ = 0°) is structurally prohibited such as the cases of disubstituted *o*-CBs.^26,69,79^ In these cases, other mechanisms such as photoinduced electron transfer between two fluorophores are more likely for fluorescence quenching.

### 3.2 Substitution Effect

On the basis of our findings, the LUMO energy level of *o*-CB and its response to structural changes in the excited state (e.g., C_1_–C_2_ bond elongation or intramolecular twist) play a key role in determining the nature and energetics of S_1_ → S_0_ transitions, and, in principle, it could be tuned by substituting different elements or groups on the carbon or boron atoms. Thus, we envisioned the investigation of varied substituents (−F, −Cl, −CN, and −OH) in the current *o*-CB–*Ant* system. It has been previously shown that different substituents on (car)borane clusters can induce strong mesomeric (±M) and inductive (±I) effects on the frontier energy levels of these clusters.^80^ In this regard, we investigated how substitution may affect the photophysical properties of *o*-CB–*Ant* by altering the LUMO energy level of the *o*-carborane cluster. In Table 1, the effects on the excited-state geometries and the transition energies are given for *o*-CB–*Ant* derivatives with different substitutions on one of the carbon atoms (see Figure S5 for the geometries of substituted derivatives).

**Table 1:** Transition Energies (*E*_0→1_ or *E*_1→0_), Oscillator Strengths (*f*), Adiabatic S_1_ State Energies (*E*_S1_), C_1_–C_2_ Lengths, and Dihedral Angles (φ) for Ground-State and Three Main S_1_ State Conformations of *o*-CB–*Ant*^a^

	S_0,min_					LE state				
substitution	*E*_0→1_	*f*	*E*_S1_	C_1_–C_2_	φ	*E*_1→0_	*f*	*E*_S1_	C_1_–C_2_	φ
H	3.37	0.22	3.37	1.65	–15	2.61	0.25	2.97	1.66	–13
F	3.31	0.23	3.31	1.69	–21	2.51	0.25	2.90	1.69	–19
Cl	3.23	0.22	3.23	1.73	–27	2.24^b^	0.21	2.77	1.73	–25
CN	3.23	0.22	3.23	1.70	–23	2.26^b^	0.21	2.78	1.70	–26
OH	3.30	0.22	3.30	1.76	–22	2.50	0.25	2.89	1.75	–21
	HLCT state					CT state				
H	2.25	0.49	2.97	2.25	–86	1.31	0.00	2.79	2.53	0
F	2.24	0.50	2.61	2.25	–87	1.21	0.00	2.36	2.51	0
Cl	2.17	0.49	2.18	2.27	–87	1.04	0.00	2.00	2.56	0
CN	2.02	0.50	2.08	2.32	–87	0.90	0.00	1.81	2.54	0
OH	2.37	0.40	2.57	2.13	–86	1.18	0.00	2.49	2.55	0

a Energies are given in eV and C_1_–C_2_ lengths are given in Å.

b LE geometry calculations for Cl and CN substituted molecules were performed with a fixed C_1_–C_2_ bond since they tend toward the formation of the HLCT state for these derivatives.

For the vertical S_0_ → S_1_ transitions (i.e., optical absorption), it is seen that substitution causes only a slight energetic red shift for all systems as compared to that with −H substituents. The reason is that these transitions mainly maintain the π → π* character localized on the anthracene unit. The red shift is 0.06–0.07 eV for −F and −OH substitutions, whereas the same red shift with −Cl and −CN substitutions are slightly more pronounced (0.14 eV). Similarly, the S_1_ → S_0_ transition (i.e., optical emission) shows a local π → π* character for all substitutions in the case of the LE state as well. However, in this case the red shifts with respect to that with −H substituents are significantly higher with −Cl and −CN substitutions (∼0.35 eV). A similar shift is also calculated for the adiabatic excited-state energies (*E*_S1_) as well. In comparison, for the twisted conformation where φ = −86 or −87°, the substitution shows a more pronounced stabilization effect on the calculated *E*_S1_. In the case of unsubstituted *o*-CB–*Ant*, there is no energy difference for *E*_S1_ values between HLCT and LE states. On the other hand, the HLCT state becomes significantly more stable upon substitution. This stabilization mainly originates from the fact that the energy difference between the minimum S_0_ geometry and the twisted-conformation S_0_ geometry (Δ*E*_0_) becomes much smaller upon substitution. The stabilization of the HLCT state when compared to the LE state is more pronounced with substitutions showing a strong −M effect (0.70 eV for −CN), while −OH and −F substitutions show ∼0.3 eV decreases for the *E*_S1_ of the twisted conformation compared to the LE conformations. We note that the CT state remains the global minimum with all substituents. For the unsubstituted system, the energy difference between CT and HLCT states is calculated to be 0.18 eV. With substitution, this energy difference becomes the largest for the case of −CN (0.27 eV), whereas it decreases by 0.08 eV for −OH substitution.

**Figure 4:**
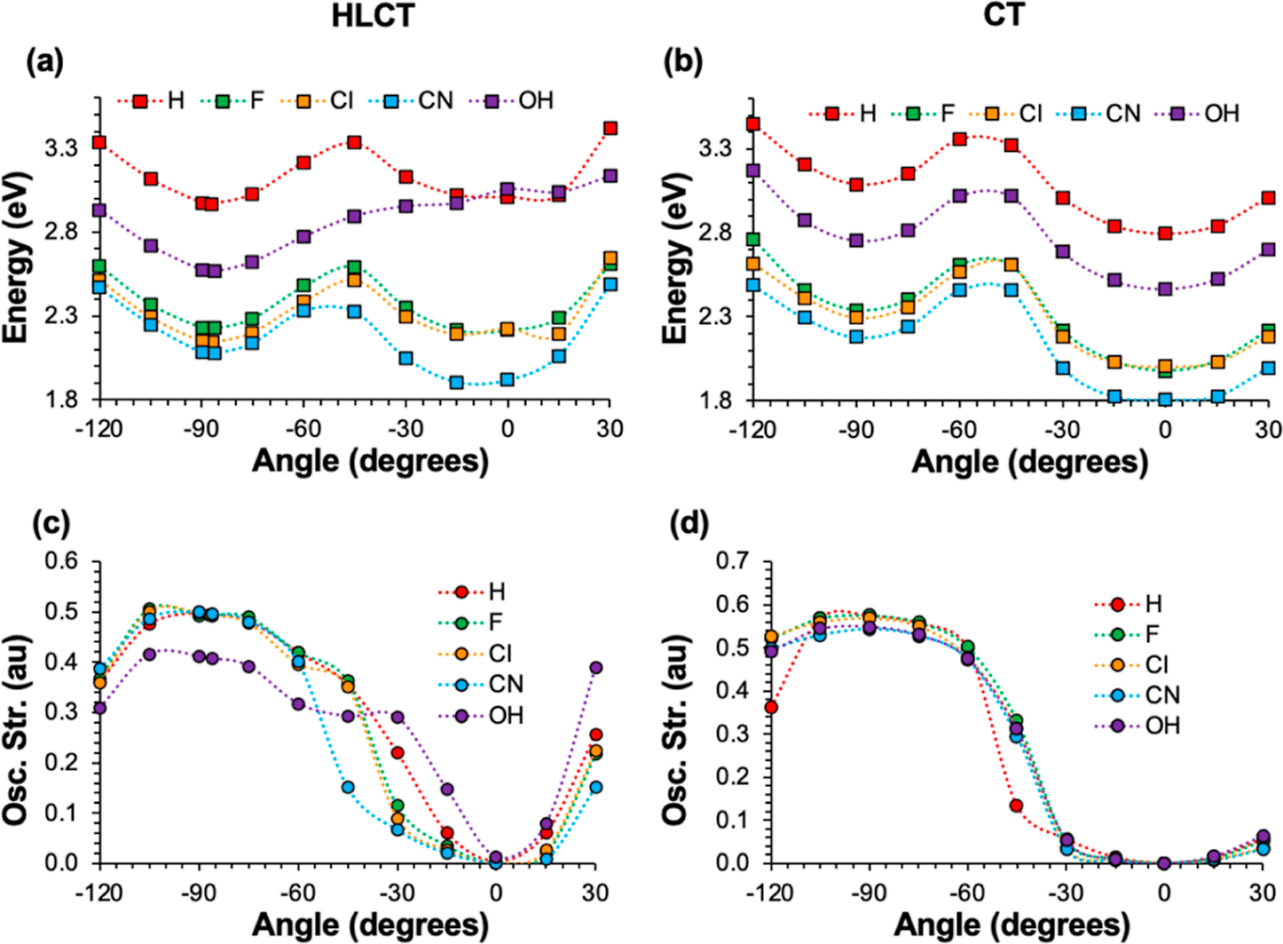
Potential energy diagrams for the rotation at fixed C_1_–C_2_ bond lengths of the (a) HLCT state and (b) CT state for the C-substituted derivatives of the *o*-*CB*–*Ant* dyad with their corresponding oscillator strengths (c,d).

In addition to the geometric parameters of fully optimized excited-state geometries, we have also scanned the PESs of the S_1_ state with respect to φ for fixed C_1_–C_2_ bond lengths for each substituted-*o*-CB–*Ant*. The comparison of *E*_S1_’s is shown in Figure 4a,b for a fixed C_1_–C_2_ bond length of partially (C_1_–C_2_ = 2.32–2.13 Å) and fully stretched (C_1_–C_2_ = 2.51–2.56 Å) conformations of each system, respectively. Figure 4c,d shows the corresponding oscillator strengths for these S_1_ → S_0_ transitions. When the C_1_–C_2_ bond lengths are partially stretched (those corresponding to the twisted conformations for each system), the calculated PESs (Figure 4a) with −Cl and −F substitutions show a similar trend to the unsubstituted case. With the −CN substitution, however, the HLCT state (φ = −87°) does not correspond to the minimum point for this PES anymore as the CT state (φ = 0°) is predicted to be 0.16 eV more stable compared to the HLCT state. This stabilization results from the strong −M effect and longer C_1_–C_2_ bond length induced by the −CN substituents, which strongly stabilizes the *o*-carborane-based orbital energy levels. In the case of −OH substitution, the HLCT state is predicted to be more stable, while the CT state is predicted to be strongly destabilized as shown in Figure 4a. For fully stretched conformations (C_1_–C_2_ = 2.51–2.56 Å, Figure 4b), all substituents show a similar PES and oscillator strength trends compared to the unsubstituted *o*-CB–*Ant*. In all cases, the CT state with φ = 0° is predicted to be the minimum, while there is another local minimum having an HLCT character predicted for φ = ∼90°. The energy difference between the CT and HLCT states on this PES is calculated to be the largest with −CN substitution (0.37 eV) and smallest with −OH substitution (0.28 eV).

Similar to the carbon-substituted molecules, we investigate the PESs of S_1_ states for boron-substituted-*o*-CB–*Ant* with mono- and deca-substitution (Figures S6 and S7). As shown in Figures S6a and S7a, the effect of boron substitution on the calculated *E*_S1_ values is significantly less pronounced compared to the case with carbon substitutions as the effect of boron substitution on calculated Δ*E*_0_ is much smaller compared to the case with carbon substitutions. In general, both the PESs and the oscillator strengths show a similar trend for boron-substituted-*o*-CB–*Ant* compared to the unsubstituted system. We also note that the CT state (φ = 0°) is slightly more stabilized compared to the HLCT state (φ = −87°) with −F, −Cl, and −CN substitutions. As a result, the CT state becomes energetically more favorable even for the partially elongated C_1_–C_2_ bond lengths (Figure S6a) with −Cl and −CN substitutions. In comparison, the energy of the CT state shows a slight increase with −OH substitution for both PESs. We also note that substituent effects, as expected, are significantly larger with deca-substitution as illustrated for the F-substituted system (Figures S6c,d and S7c,d). Based on our results with carbon and boron substitutions, it is revealed that both CT and HLCT states become energetically more stable compared to LE state when the substituent shows an “–M effect” (e.g., −CN), whereas substituents showing “+M effects” (e.g., −OH) can result in an energy increase for the CT state, especially for partially stretched C_1_–C_2_ bond lengths.

### 3.3 Effect of the Fluorophore

**Figure 5:**
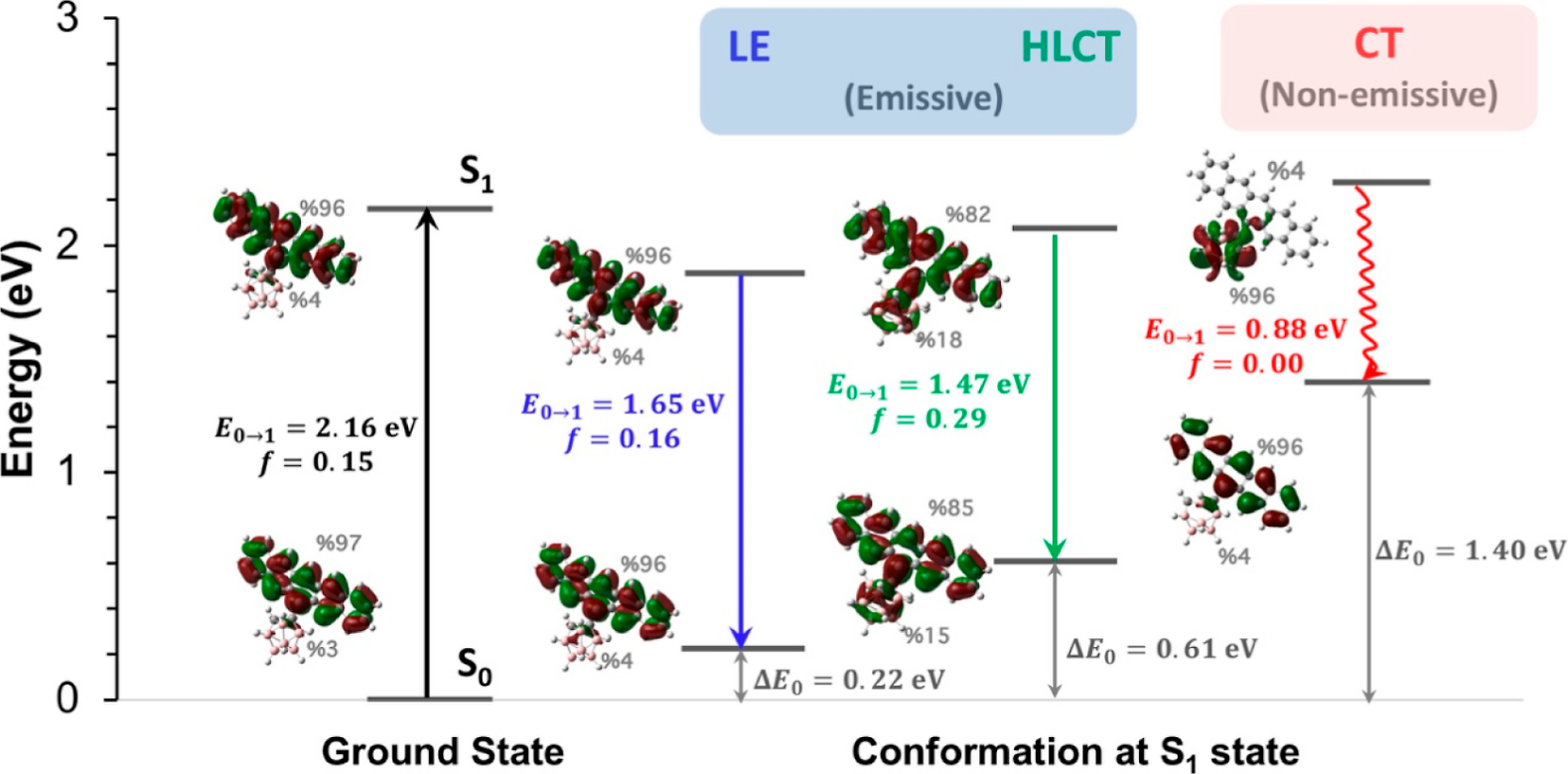
Illustration of the absorption and potential emission paths along with the corresponding geometries of the o-*CB*–*Pnt* system. The conformations for LE and CT states correspond to the minimum energy points on the S_1_ PES, while the HLCT state is obtained with excited-state geometry optimization with constraints (C_1_–C_2_ = 2.25 Å and φ = −87°). % contributions of pentacene and o-*CB*-based orbitals to HOMO and LUMO for each transition are shown in the orbital pictures.

**Figure 6:**
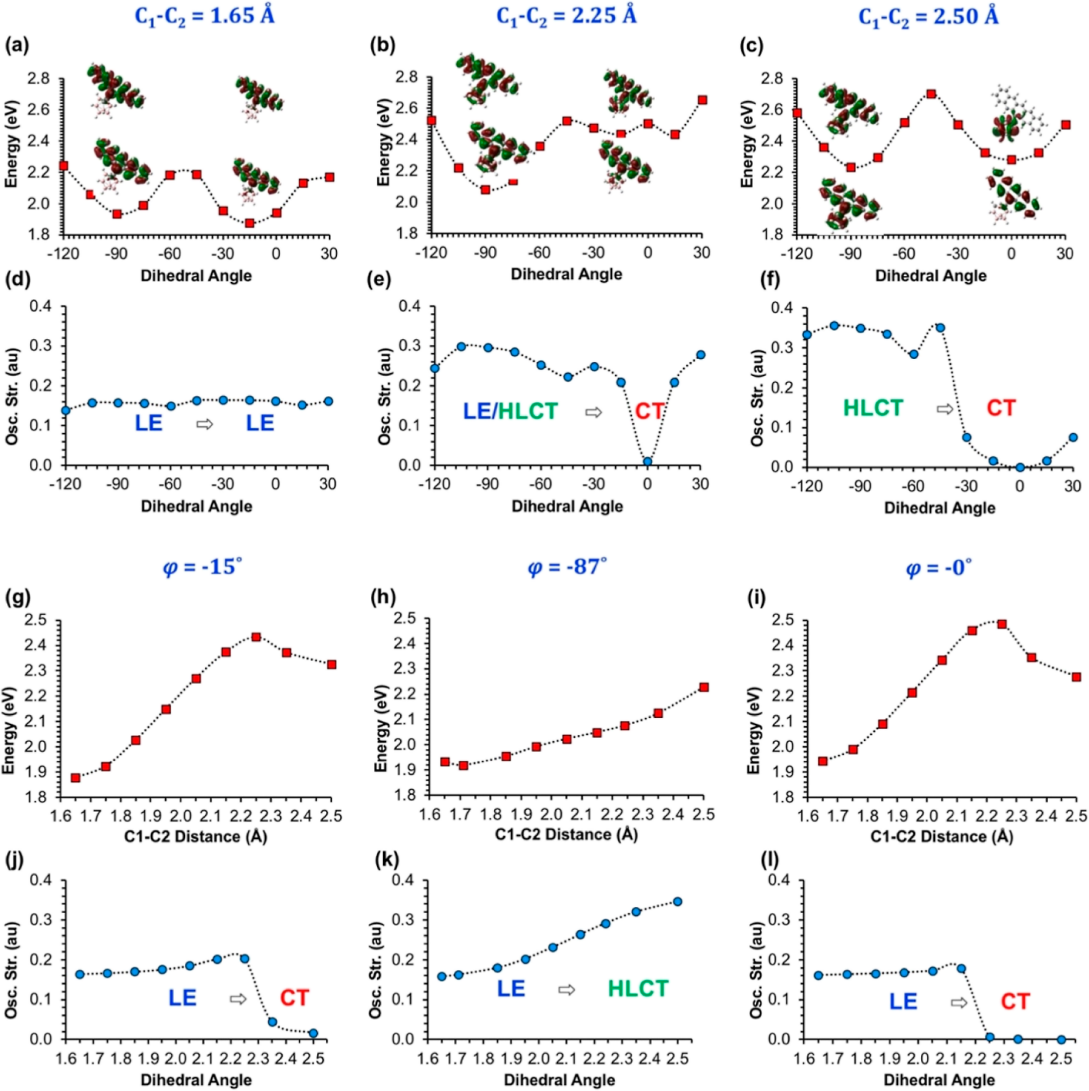
PESs for the adiabatic excited-state energies (*E*_S1_) with respect to φ at fixed C_1_–C_2_ bond lengths (a–c) and calculated oscillator strengths for the corresponding S_1_ → S_0_ (d–f). The same PESs (g–i) and oscillator strengths (j–l) are given with respect to C_1_–C_2_ bond lengths at a fixed φ as well.

To investigate how the energy levels of the conjugated π-system may affect the photoexcitation processes for *o*-CB–*fluorophore* systems, an anthracene unit was replaced with a π-extended fused acene molecule, pentacene. Energies of the vertical S_0_ → S_1_ transition and possible S_1_ → S_0_ pathways for the *o*-CB-pentacene (*o*-CB–*Pnt*) molecule are shown along with the excited-state characteristics in Figure 5. As expected, both the vertical S_0_ → S_1_ and S_1_ → S_0_ transitions in the LE state occur via π → π* transitions. In addition, the LE state exhibits a very similar geometry compared to the ground-state conformation. To see the effect of the partially elongated C_1_–C_2_ bond length and twisted geometry, we perform an excited-state geometry optimization with constraints (C_1_–C_2_ = 2.25 Å and φ = −87°), which corresponds to the local minimum conformation for the HLCT state of *o*-CB–*Ant*. A moderate increase in the oscillator strength is observed for the S_1_ → S_0_ transition for this conformer; however, unlike the case in the *o*-CB–*Ant* system, the adiabatic energy of the excited state (*E*_S1_) for this conformation shows an increase compared to the same energy for the LE state (1.88 eV → 2.08 eV) in the case of *o*-CB–*Pnt*. In this twisted conformation, the contribution of the *o*-carborane-based orbitals only slightly increases, in a similar extent for both HOMO and LUMO levels. As a result, the S_1_ → S_0_ transition shows an LE dominant HLCT character for this conformation in contrast to the twisted conformer of *o*-CB–*Ant*. We should note that this HLCT state shown in Figure 5 does not correspond to the minimum point on the S_1_ PES for twisted conformations (φ ≈ −90°) as shown in PES in Figure 6 (vide infra). In fact, the C_1_–C_2_ bond length was found to be 1.71 Å as a result of the full S_1_ optimization of the *o*-CB–*Pnt* with an initial twisted geometry, which also exhibits a strong LE character with similar orbital contributions compared to the conformer of the LE state shown in Figure 5. Here, it is clearly seen that the longer π-conjugation and, consequently, the lower LUMO energy of pentacene (Figure S8) does not allow an efficient mixing between *o*-CB and pentacene orbitals, resulting in an absence of emission from the HLCT state. Further elongation of the C_1_–C_2_ bond length up to 2.50 Å, along with small φ, results in a third local minimum point having a pure CT character with vanishing oscillator strength for *o*-CB–*Pnt* as well. Similar to the case in *o*-CB–*Ant*, the CT state shows the minimum transition energy (0.88 eV) among the three excited-state conformers. However, unlike the case in *o*-CB–*Ant*, this conformation has a significantly larger *E*_S1_ compared to the other two possible conformations, showing that the relative energies of CT, HLCT, and LE states depend strongly on the energy of the fluorophore LUMO level for such systems.

Finally, Figure 6 shows the PESs for the S_1_ state of *o*-CB–*Pnt* with respect to changing φ values (Figure 6a–c) and C_1_–C_2_ bond lengths (Figure 6g–l), along with the corresponding oscillator strengths for the S_1_ → S_0_ transitions (Figure 6d–f,j–l, respectively). For C_1_–C_2_ = 1.65 Å, it is seen that calculated PES and the excited-state character of S_1_ → S_0_ transitions with respect to changing φ values are quite similar to the case in *o*-CB–*Ant*. Similar to *o*-CB–*Ant* system, the S_1_ → S_0_ transitions mainly originate from π–π* transition on the pentacene π-system for this PES; therefore, the excited-state characters and the oscillator strengths do not exhibit a large change through the rotation at this bond length. Interestingly, S_1_ → S_0_ transitions mainly stay as an LE dominant HLCT transition when the C_1_–C_2_ bond length is 2.25 Å, except for φ = 0°. For φ = 0°, the excited-state character shows a sharp LE to CT transition; however, the calculated *E*_S1_ do not show a local minimum around this φ value. These differences between the calculated PESs of *o*-CB–*Ant* and *o*-CB–*Pnt* originate from the fact that the mixing of pentacene- and *o*-carborane-based levels for the LUMO still remains energetically unfavorable for this C_1_–C_2_ bond length, unlike the case in *o*-CB–*Ant*. On the other hand, when C_1_–C_2_ is 2.50 Å, the pentacene- and carborane-based levels show significant mixing for the LUMOs. For this bond length, the calculated PES and the oscillator strengths show a somewhat similar profile to the case in *o*-CB–*Ant*, except for the fact that even with the elongated C_1_–C_2_ bond, the CT state does not become the single energy-minimum point for this PES.

Similar to the change in φ’s, the calculated PESs and the oscillator strengths in *o*-CB–*Pnt* with respect to changing C_1_–C_2_ bond lengths also exhibit critical differences compared to the same case in *o*-CB–*Ant* (Figure 6g–l and 6j–l). For φ = 87°, the S_1_ state shows an increasing HLCT character with increasing C_1_–C_2_ bond length as evident from the calculated oscillator strengths. However, the calculated *E*_S1_ exhibits a continuous increase with increasing C_1_–C_2_ bond length, whereas the same PES remains rather flat for *o*-CB–*Ant*. As expected from the MO analysis (Figure S8), the LE → CT transition occurs in longer bond lengths for *o*-CB–*Pnt* when φ is closer to 0°. Interestingly, the energy of the S_1_ state decreases with C_1_–C_2_ bond elongation after the LE → CT transition is much less pronounced for *o*-CB–*Pnt*, as compared to the case in *o*-CB–*Ant* (Figure 3c). As a result, there is a ∼0.4 eV difference between the minimum points of LE and CT states, where the LE state is energetically more favorable. Considering the aforementioned importance of CT conformation on emission quenching, it is likely that a larger quantum yield might be expected for the *o*-CB–*Pnt* system due to the energy penalty for the CT state formation in this system.

## 4 Conclusions

In summary, we have studied the PES of the S_1_ state for *o*-carborane–anthracene (*o*-CB–*Ant*) using TDDFT methods, with a focus on the nature and energetics of S_1_ → S_0_ transitions with respect to the C_1_–C_2_ bond length in *o*-CB and dihedral angle between *o*-CB and *Ant* moieties. Furthermore, we have evaluated the effect of different substituents (F, Cl, CN, and OH) attached to carbon or boron atoms in *o*-CB, along with a π-extended acene-based fluorophore, pentacene, on the S_1_ PES and the resulting photophysical properties. In addition to the emissive LE and HLCT states which correspond to local minimum conformations on S_1_ PES, our results show the presence of a dark CT state for *o*-CB–*Ant* as a result of significant C_1_–C_2_ bond elongation (C_1_–C_2_ = 2.53 Å) in the *o*-CB moiety, which also corresponds to the lowest-energy excited state on the S_1_ PES in our investigation. Calculated energy barriers with respect to twist angle (φ) or C_1_–C_2_ bond length are within 0.3–0.4 eV, and for the twisted conformations, the C_1_–C_2_ bond elongation is shown to occur without an energy penalty, indicating that this CT state is energetically accessible on the S_1_ surface. These results suggest that the CT state could be an important pathway on the fluorescence quenching mechanism of *o*-CB–*Ant* and other *o*-CB–*fluorophore* systems with similar structures.

Upon carbon- or boron-substitution on *o*-*CB* with substituents showing a strong -M effect such as −CN, both the CT and HLCT states become energetically even more favorable compared to the LE state; however, substituents showing “+M effects” (e.g., −OH) can result in an energy increase for the CT state, especially for partially stretched C_1_–C_2_ bond lengths. This result mainly originates from tuning the LUMO energy level of *o*-CB, which affects the energetics of CT between two moieties. Furthermore, it is shown that the LUMO energy of the fluorophore is also critical for the relative energies of CT, HLCT, and LE states and for the calculated energy barriers on the S_1_ PES. When anthracene is replaced with π-extended pentacene as the fluorophore (*o*-CB–*Pnt*), the CT state is no longer predicted as the minimum-energy point on S_1_ PES as a result of the lower LUMO energy level of *Pnt*, and the calculated energy barriers for C_1_–C_2_ bond elongation show a considerable increase (0.5–0.6 eV). Our results clearly emphasize that the energetics of emissive and non-emissive transitions along with the energy barriers on the S_1_ PES can be tuned with respect to substituents or fluorophore energy levels in *o*-CB–*fluorophore* systems, which is expected to guide future experimental work in emissive *o*-CB–*fluorophore* systems and their sensing/optoelectronic applications.

## Supporting Information Available

Geometric parameters for *o*-CB–*Ant*, benchmark results for the emission and absorption energies, frontier orbitals of the three conformations for the S_1_ state of *o*-CB–*Ant*, Δ*r* and ∧ values for the excited states of *o*-CB–*Ant*, atomic labels for the heat maps, heat maps of TDM graphs for S_1_ states, relative energies of the frontier levels for *o*-CB and *Ant* moieties, positions of substituents for *o*-CB–*Ant* derivatives, PESs for the B-substituted derivatives of the *o*-CB–*Ant*, and relative energies of the frontier levels for *o*-CB and *Pnt* moieties (PDF)

## References

[ref1] Mei J.; Leung N. L. C.; Kwok R. T. K.; Lam J. W. Y.; Tang B. Z. Aggregation-Induced Emission: Together We Shine, United We Soar!. Chem. Rev. 2015, 115, 11718–11940. 10.1021/acs.chemrev.5b00263. 26492387

[ref2] Ostroverkhova O. Organic Optoelectronic Materials: Mechanisms and Applications. Chem. Rev. 2016, 116, 13279–13412. 10.1021/acs.chemrev.6b00127. 27723323

[ref3] Lin Z.; Kabe R.; Wang K.; Adachi C. Influence of Energy Gap between Charge-Transfer and Locally Excited States on Organic Long Persistence Luminescence. Nat. Commun. 2020, 11, 191 10.1038/s41467-019-14035-y. 31924793 PMC6954229

[ref4] Cao D.; Liu Z.; Verwilst P.; Koo S.; Jangjili P.; Kim J. S.; Lin W. Coumarin-Based Small-Molecule Fluorescent Chemosensors. Chem. Rev. 2019, 119, 10403–10519. 10.1021/acs.chemrev.9b00145. 31314507

[ref5] Dong Y. Q.; Lam J. W. Y.; Tang B. Z. Mechanochromic Luminescence of Aggregation-Induced Emission Luminogens. J. Phys. Chem. Lett. 2015, 6, 3429–3436. 10.1021/acs.jpclett.5b01090. 26268912

[ref6] Sasaki S.; Drummen G. P. C.; Konishi G.-i. Recent Advances in Twisted Intramolecular Charge Transfer (TICT) Fluorescence and Related Phenomena in Materials Chemistry. J. Mater. Chem. C 2016, 4, 2731–2743. 10.1039/c5tc03933a.

[ref7] Usta H.; Alimli D.; Ozdemir R.; Dabak S.; Zorlu Y.; Alkan F.; Tekin E.; Can A. Highly Efficient Deep-Blue Electroluminescence Based on a Solution-Processable A−π–D−π–A Oligo (p -Phenyleneethynylene) Small Molecule. ACS Appl. Mater. Interfaces 2019, 11, 44474–44486. 10.1021/acsami.9b12971. 31609580

[ref8] Phillips A. T.; Yu Z.; Stewart D. J.; Cooper T. M.; Haley J. E.; Tan L.-S.; Grusenmeyer T. A. Influence of Structural Isomerism on the Photophysical Properties of a Series of Donor–Acceptor 1-Naphthalenecarbonitrile Derivatives Possessing Amine Substituents. J. Phys. Chem. A 2020, 124, 2113–2122. 10.1021/acs.jpca.9b10788. 32068405

[ref9] Wade K. The Structural Significance of the Number of Skeletal Bonding Electron-Pairs in Carboranes, the Higher Boranes and Borane Anions, and Various Transition-Metal Carbonyl Cluster Compounds. J. Chem. Soc., Chem. Commun. 1971, 9, 792 10.1039/c29710000792.

[ref10] Mingos D. M. P. Polyhedral Skeletal Electron Pair Approach. Acc. Chem. Res. 1984, 17, 311–319. 10.1021/ar00105a003.

[ref11] Welch A. J. The Significance and Impact of Wade’s Rules. Chem. Commun. 2013, 49, 3615–3616. 10.1039/c3cc00069a. 23535980

[ref12] Okotrub A. V.; Bulusheva L. G.; Volkov V. V. Electron Interactions in the Closo-Carboranes 1,2- and 1,7-C_2_B_10_H_12_. J. Mol. Struct. 2000, 520, 33–38. 10.1016/s0022-2860(99)00310-5.

[ref13] Tsuboya N.; Lamrani M.; Hamasaki R.; Ito M.; Mitsuishi M.; Miyashita T.; Yamamoto Y. Nonlinear Optical Properties of Novel Carborane-Ferrocene Conjugated Dyads. Electron-Withdrawing Characteristics of Carboranes. J. Mater. Chem. 2002, 12, 2701–2705. 10.1039/b202236b.

[ref14] Crespo O.; Gimeno M. C.; Laguna A.; Ospino I.; Aullón G.; Oliva J. M. Organometallic Gold Complexes of Carborane. Theoretical Comparative Analysis of Ortho, Meta, and Para Derivatives and Luminescence Studies. Dalton Trans. 2009, 3807–3813. 10.1039/b820803d. 19417947

[ref15] Hosmane N. S. Boron Science; CRC Press, 2016.

[ref16] Armstrong A. F.; Valliant J. F. The Bioinorganic and Medicinal Chemistry of Carboranes: From New Drug Discovery to Molecular Imaging and Therapy. Dalton Trans. 2007, 4240–4251. 10.1039/b709843j. 17893811

[ref17] Grimes R. N. Carboranes, 3rd ed.; Academic Press, 2016.

[ref18] Grimes R. N. Carboranes in the Chemist’s Toolbox. Dalton Trans. 2015, 44, 5939–5956. 10.1039/c5dt00231a. 25710453

[ref19] Barth R. F.; Mi P.; Yang W. Boron Delivery Agents for Neutron Capture Therapy of Cancer. Cancer Commun. 2018, 38, 35 10.1186/s40880-018-0299-7. PMC6006782 29914561

[ref20] Scholz M.; Hey-Hawkins E. Carbaboranes as Pharmacophores: Properties, Synthesis, and Application Strategies. Chem. Rev. 2011, 111, 7035–7062. 10.1021/cr200038x. 21780840

[ref21] Núñez R.; Tarrés M.; Ferrer-Ugalde A.; de Biani F. F.; Teixidor F. Electrochemistry and Photoluminescence of Icosahedral Carboranes, Boranes, Metallacarboranes, and Their Derivatives. Chem. Rev. 2016, 116, 14307–14378. 10.1021/acs.chemrev.6b00198. 27960264

[ref22] Naito H.; Nishino K.; Morisaki Y.; Tanaka K.; Chujo Y. Highly-Efficient Solid-State Emissions of Anthracene–o-Carborane Dyads with Various Substituents and Their Thermochromic Luminescence Properties. J. Mater. Chem. C 2017, 5, 10047–10054. 10.1039/C7TC02682J.

[ref23] Axtell J. C.; Saleh L. M. A.; Qian E. A.; Wixtrom A. I.; Spokoyny A. M. Synthesis and Applications of Perfunctionalized Boron Clusters. Inorg. Chem. 2018, 57, 2333–2350. 10.1021/acs.inorgchem.7b02912. 29465227 PMC5985200

[ref24] Naito H.; Nishino K.; Morisaki Y.; Tanaka K.; Chujo Y. Solid-State Emission of the Anthracene- o -Carborane Dyad from the Twisted-Intramolecular Charge Transfer in the Crystalline State. Angew. Chem., Int. Ed. 2017, 56, 254–259. 10.1002/anie.201609656. 27911472

[ref25] Naito H.; Morisaki Y.; Chujo Y. O-Carborane-Based Anthracene: A Variety of Emission Behaviors. Angew. Chem., Int. Ed. 2015, 54, 5084–5087. 10.1002/anie.201500129. 25729004

[ref26] Ferrer-Ugalde A.; González-Campo A.; Viñas C.; Rodríguez-Romero J.; Santillan R.; Farfán N.; Sillanpää R.; Sousa-Pedrares A.; Núñez R.; Teixidor F. Fluorescence of New O-Carborane Compounds with Different Fluorophores: Can It Be Tuned?. Chem.—Eur. J. 2014, 20, 9940–9951. 10.1002/chem.201402396. 24976049

[ref27] Martin K. L.; Krishnamurthy A.; Strahan J.; Young E. R.; Carter K. R. Excited State Characterization of Carborane-Containing Poly(Dihexyl Fluorene)s. J. Phys. Chem. A 2019, 123, 1701–1709. 10.1021/acs.jpca.8b07955. 30608152

[ref28] Jin G. F.; Cho Y.-J.; Wee K.-R.; Hong S. A.; Suh I.-H.; Son H.-J.; Lee J.-D.; Han W.-S.; Cho D. W.; Kang S. O. BODIPY Functionalized o-Carborane Dyads for Low-Energy Photosensitization. Dalton Trans. 2015, 44, 2780–2787. 10.1039/c4dt03123g. 25482506

[ref29] Chaari M.; Kelemen Z.; Choquesillo-Lazarte D.; Gaztelumendi N.; Teixidor F.; Viñas C.; Nogués C.; Núñez R. Efficient Blue Light Emitting Materials Based on m -Carborane–Anthracene Dyads. Structure, Photophysics and Bioimaging Studies. Biomater. Sci. 2019, 7, 5324–5337. 10.1039/c9bm00903e. 31620701

[ref30] Chaari M.; Kelemen Z.; Planas J. G.; Teixidor F.; Choquesillo-Lazarte D.; ben Salah A.; Viñas C.; Núñez R. Photoluminescence in m-Carborane–Anthracene Triads: A Combined Experimental and Computational Study. J. Mater. Chem. C 2018, 6, 11336–11347. 10.1039/c8tc03741h.

[ref31] Chaari M.; Kelemen Z.; Choquesillo-Lazarte D.; Teixidor F.; Viñas C.; Núñez R. Anthracene–Styrene-Substituted m -Carborane Derivatives: Insights into the Electronic and Structural Effects of Substituents on Photoluminescence. Inorg. Chem. Front. 2020, 7, 2370–2380. 10.1039/d0qi00127a.

[ref32] Gon M.; Tanaka K.; Chujo Y. Concept of Excitation-Driven Boron Complexes and Their Applications for Functional Luminescent Materials. Bull. Chem. Soc. Jpn. 2019, 92, 7–18. 10.1246/bcsj.20180245.

[ref33] You D. K.; Lee J. H.; Choi B. H.; Hwang H.; Lee M. H.; Lee K. M.; Park M. H. Effects of Multi-Carborane Substitution on the Photophysical and Electron-Accepting Properties of o-Carboranylbenzene Compounds. Eur. J. Inorg. Chem. 2017, 2017, 2496–2503. 10.1002/ejic.201700192.

[ref34] Ferrer-Ugalde A.; Cabrera-González J.; Juárez-Pérez E. J.; Teixidor F.; Pérez-Inestrosa E.; Montenegro J. M.; Sillanpää R.; Haukka M.; Núñez R. Carborane-Stilbene Dyads: The Influence of Substituents and Cluster Isomers on Photoluminescence Properties. Dalton Trans. 2017, 46, 2091–2104. 10.1039/c6dt04003a. 28045166

[ref35] Tominaga M.; Naito H.; Morisaki Y.; Chujo Y. Control of the Emission Behaviors of Trifunctional O-Carborane Dyes. Asian J. Org. Chem. 2014, 3, 624–631. 10.1002/ajoc.201300280.

[ref36] González-Campo A.; Ferrer-Ugalde A.; Viñas C.; Teixidor F.; Sillanpää R.; Rodríguez-Romero J.; Santillan R.; Farfán N.; Núñez R. A Versatile Methodology for the Controlled Synthesis of Photoluminescent High-Boron-Content Dendrimers. Chem.—Eur. J. 2013, 19, 6299–6312. 10.1002/chem.201203771. 23494750

[ref37] Kokado K.; Chujo Y. Multicolor Tuning of Aggregation-Induced Emission through Substituent Variation of Diphenyl-o-Carborane. J. Org. Chem. 2011, 76, 316–319. 10.1021/jo101999b. 21158383

[ref38] Kokado K.; Chujo Y. Emission via Aggregation of Alternating Polymers with O-Carborane and p-Phenylene-Ethynylene Sequences. Macromolecules 2009, 42, 1418–1420. 10.1021/ma8027358.

[ref39] Kim S.-Y.; Cho Y.-J.; Son H.-J.; Cho D. W.; Kang S. O. Photoinduced Electron Transfer in a BODIPY- Ortho -Carborane Dyad Investigated by Time-Resolved Transient Absorption Spectroscopy. J. Phys. Chem. A 2018, 122, 3391–3397. 10.1021/acs.jpca.8b01539. 29554419

[ref40] Marsh A. v.; Dyson M. J.; Cheetham N. J.; Bidwell M.; Little M.; White A. J. P.; Warriner C. N.; Swain A. C.; McCulloch I.; Stavrinou P. N.; Meskers S. C. J.; Heeney M. Correlating the Structural and Photophysical Properties of Ortho, Meta, and Para-Carboranyl–Anthracene Dyads. Adv. Electron. Mater. 2020, 6, 2000312 10.1002/aelm.202000312.

[ref41] Weber L.; Kahlert J.; Brockhinke R.; Böhling L.; Brockhinke A.; Stammler H. G.; Neumann B.; Harder R. A.; Fox M. A. Luminescence Properties of C-Diazaborolyl-Ortho-Carboranes as Donor-Acceptor Systems. Chem.—Eur. J. 2012, 18, 8347–8357. 10.1002/chem.201200390. 22623079

[ref42] Nishino K.; Yamamoto H.; Tanaka K.; Chujo Y. Development of Solid-State Emissive Materials Based on Multifunctional o-Carborane-Pyrene Dyads. Org. Lett. 2016, 18, 4064–4067. 10.1021/acs.orglett.6b01920. 27471860

[ref43] Tanaka K.; Nishino K.; Ito S.; Yamane H.; Suenaga K.; Hashimoto K.; Chujo Y. Development of Solid-State Emissive o-Carboranes and Theoretical Investigation of the Mechanism of the Aggregation-Induced Emission Behaviors of Organoboron “Element-Blocks.”. Faraday Discuss. 2017, 196, 31–42. 10.1039/c6fd00155f. 27900376

[ref44] Furue R.; Nishimoto T.; Park I. S.; Lee J.; Yasuda T. Aggregation-Induced Delayed Fluorescence Based on Donor/Acceptor-Tethered Janus Carborane Triads: Unique Photophysical Properties of Nondoped OLEDs. Angew. Chem., Int. Ed. 2016, 55, 7171–7175. 10.1002/anie.201603232. 27145481

[ref45] Ochi J.; Tanaka K.; Chujo Y. Experimental Proof for Emission Annihilation through Bond Elongation at the Carbon-Carbon Bond in: O -Carborane with Fused Biphenyl-Substituted Compounds. Dalton Trans. 2021, 50, 1025–1033. 10.1039/d0dt03618h. 33367426

[ref46] Wu X.; Guo J.; Cao Y.; Zhao J.; Jia W.; Chen Y.; Jia D. Mechanically Triggered Reversible Stepwise Tricolor Switching and Thermochromism of Anthracene- o -Carborane Dyad. Chem. Sci. 2018, 9, 5270–5277. 10.1039/c8sc00833g. 29997882 PMC6001385

[ref47] Kim S.; Lee J. H.; So H.; Ryu J.; Lee J.; Hwang H.; Kim Y.; Park M. H.; Lee K. M. Spirobifluorene-Based o-Carboranyl Compounds: Insights into the Rotational Effect of Carborane Cages on Photoluminescence. Chem.—Eur. J. 2020, 26, 548–557. 10.1002/chem.201904491. 31657858

[ref48] Wee K.-R.; Cho Y.-J.; Song J. K.; Kang S. O. Multiple Photoluminescence from 1,2-Dinaphthyl-Ortho-Carborane. Angew. Chem., Int. Ed. 2013, 52, 9682–9685. 10.1002/anie.201304321. 23881691

[ref49] Marsh A. v.; Cheetham N. J.; Little M.; Dyson M.; White A. J. P.; Beavis P.; Warriner C. N.; Swain A. C.; Stavrinou P. N.; Heeney M. Carborane-Induced Excimer Emission of Severely Twisted Bis-o-Carboranyl Chrysene. Angew. Chem., Int. Ed. 2018, 57, 10640–10645. 10.1002/anie.201805967. PMC6099267 29952051

[ref50] Nishino K.; Yamamoto H.; Tanaka K.; Chujo Y. Solid-State Thermochromic Luminescence through Twisted Intramolecular Charge Transfer and Excimer Formation of a Carborane–Pyrene Dyad with an Ethynyl Spacer. Asian J. Org. Chem. 2017, 6, 1818–1822. 10.1002/ajoc.201700390.

[ref51] Londesborough M. G. S.; Dolanský J.; Cerdán L.; Lang K.; Jelínek T.; Oliva J. M.; Hnyk D.; Roca-Sanjuán D.; Francés-Monerris A.; Martinčík J.; Nikl M.; Kennedy J. D. Thermochromic Fluorescence from B_18_H_20_(NC_5_H_5_)_2_: An Inorganic-Organic Composite Luminescent Compound with an Unusual Molecular Geometry. Adv. Opt. Mater. 2017, 5, 1600694 10.1002/adom.201600694.

[ref52] Duan Y. C.; Pan Q. Q.; Zhao Z. W.; Gao Y.; Wu Y.; Zhao L.; Geng Y.; Zhang M.; Su Z. M. Theoretical Simulations of Thermochromic and Aggregation-Induced Emission Behaviors of a Series of Red-Light Anthracene- o- Carborane Derivatives. Chem.—Eur. J. 2021, 27, 9571–9579. 10.1002/chem.202100235. 33786898

[ref53] Frisch M. J.; Trucks G. W.; Schlegel H. B.; Scuseria G. E.; Robb M. A.; Cheeseman J. R.; Scalmani G.; Barone V.; Petersson G. A.; Nakatsuji H. Gaussian 09; Gaussian, Inc.: Wallingford CT, 2016.

[ref54] Mennucci B. Polarizable Continuum Model. Wiley Interdiscip. Rev.: Comput. Mol. Sci. 2012, 2, 386–404. 10.1002/wcms.1086.

[ref55] Tomasi J.; Mennucci B.; Cammi R. Quantum Mechanical Continuum Solvation Models. Chem. Rev. 2005, 105, 2999–3094. 10.1021/cr9904009. 16092826

[ref56] Cancès E.; Mennucci B. Comment on “Reaction Field Treatment of Charge Penetration” [J. Chem. Phys. 112, 5558 (2000)]. J. Chem. Phys. 2001, 114, 4744 10.1063/1.1349091.

[ref57] Pascual-Ahuir J. L.; Silla E.; Tomasi J.; Bonaccorsi R. Electrostatic Interaction of a Solute with a Continuum. Improved Description of the Cavity and of the Surface Cavity Bound Charge Distribution. J. Comput. Chem. 1987, 8, 778–787. 10.1002/jcc.540080605.

[ref58] Miertuš S.; Scrocco E.; Tomasi J. Electrostatic Interaction of a Solute with a Continuum. A Direct Utilizaion of AB Initio Molecular Potentials for the Prevision of Solvent Effects. J. Chem. Phys. 1981, 55, 117–129. 10.1016/0301-0104(81)85090-2.

[ref59] Dennington R. D.; Keith T. A.; Millam J. M. GaussView; Semichem, Inc.: Wallingford CT, 2009.

[ref60] O’boyle N. M.; Tenderholt A. L.; Langner K. M. Cclib: A Library for Package-Independent Computational Chemistry Algorithms. J. Comput. Chem. 2008, 29, 839–845. 10.1002/jcc.20823. 17849392

[ref61] Lu T.; Chen F. Multiwfn: A Multifunctional Wavefunction Analyzer. J. Comput. Chem. 2012, 33, 580–592. 10.1002/jcc.22885. 22162017

[ref62] Ji L.; Riese S.; Schmiedel A.; Holzapfel M.; Fest M.; Nitsch J.; Curchod B. F. E.; Friedrich A.; Wu L.; al Mamari H. H.; Hammer S.; Pflaum J.; Fox M. A.; Tozer D. J.; Finze M.; Lambert C.; Marder T. B. Thermodynamic Equilibrium between Locally Excited and Charge-Transfer States through Thermally Activated Charge Transfer in 1-(Pyren-2′-yl)- o -Carborane. Chem. Sci. 2022, 13, 5205–5219. 10.1039/d1sc06867a. 35655553 PMC9093154

[ref63] Perdew J. P. Density-Functional Approximation for the Correlation Energy of the Inhomogeneous Electron Gas. Phys. Rev. B: Condens. Matter Mater. Phys. 1986, 33, 8822–8824. 10.1103/physrevb.33.8822. 9938299

[ref64] Becke A. D. Density-Functional Exchange-Energy Approximation with Correct Asymptotic Behavior. Phys. Rev. A 1988, 38, 3098–3100. 10.1103/physreva.38.3098. 9900728

[ref65] Becke A. D. Density-Functional Thermochemistry. III. The Role of Exact Exchange. J. Chem. Phys. 1993, 98, 5648–5652. 10.1063/1.464913.

[ref66] Yanai T.; Tew D. P.; Handy N. C. A New Hybrid Exchange-Correlation Functional Using the Coulomb-Attenuating Method (CAM-B3LYP). Chem. Phys. Lett. 2004, 393, 51–57. 10.1016/j.cplett.2004.06.011.

[ref67] Zhao Y.; Truhlar D. G. The M06 Suite of Density Functionals for Main Group Thermochemistry, Thermochemical Kinetics, Noncovalent Interactions, Excited States, and Transition Elements: Two New Functionals and Systematic Testing of Four M06-Class Functionals and 12 Other Function. Theor. Chem. Acc. 2008, 120, 215–241. 10.1007/s00214-007-0310-x.

[ref68] Kim S.; Lee J. H.; So H.; Kim M.; Mun M. S.; Hwang H.; Park M. H.; Lee K. M. Insights into the Effects of Substitution Position on the Photophysics of Mono-: O -Carborane-Substituted Pyrenes. Inorg. Chem. Front. 2020, 7, 2949–2959. 10.1039/d0qi00563k.

[ref69] Shida N.; Owaki S.; Eguchi H.; Nishikawa T.; Tomita I.; Inagi S. Bis(Pentafluorophenyl)-: O-Carborane and Its Arylthio Derivatives: Synthesis, Electrochemistry and Optical Properties. Dalton Trans. 2020, 49, 12985–12989. 10.1039/d0dt02205e. 32813754

[ref70] Li J.; Yang C.; Peng X.; Chen Y.; Qi Q.; Luo X.; Lai W.-Y.; Huang W. Stimuli-Responsive Solid-State Emission from o -Carborane–Tetraphenylethene Dyads Induced by Twisted Intramolecular Charge Transfer in the Crystalline State. J. Mater. Chem. C 2018, 6, 19–28. 10.1039/c7tc03780e.

[ref71] Plötner J.; Tozer D. J.; Dreuw A. Dependence of Excited State Potential Energy Surfaces on the Spatial Overlap of the Kohn–Sham Orbitals and the Amount of Nonlocal Hartree–Fock Exchange in Time-Dependent Density Functional Theory. J. Chem. Theory Comput. 2010, 6, 2315–2324. 10.1021/ct1001973. 26613488

[ref72] Li R.; Zheng J.; Truhlar D. G. Density Functional Approximations for Charge Transfer Excitations with Intermediate Spatial Overlap. Phys. Chem. Chem. Phys. 2010, 12, 12697 10.1039/c0cp00549e. 20733991

[ref73] Loos P.-F.; Comin M.; Blase X.; Jacquemin D. Reference Energies for Intramolecular Charge-Transfer Excitations. J. Chem. Theory Comput. 2021, 17, 3666–3686. 10.1021/acs.jctc.1c00226. 33955742

[ref74] Peach M. J. G.; Benfield P.; Helgaker T.; Tozer D. J. Excitation Energies in Density Functional Theory: An Evaluation and a Diagnostic Test. J. Chem. Phys. 2008, 128, 044118 10.1063/1.2831900. 18247941

[ref75] Laurent A. D.; Jacquemin D. TD-DFT Benchmarks: A Review. Int. J. Quantum Chem. 2013, 113, 2019–2039. 10.1002/qua.24438.

[ref76] Walker M.; Harvey A. J. A.; Sen A.; Dessent C. E. H. Performance of M06, M06-2X, and M06-HF Density Functionals for Conformationally Flexible Anionic Clusters: M06 Functionals Perform Better than B3LYP for a Model System with Dispersion and Ionic Hydrogen-Bonding Interactions. J. Phys. Chem. A 2013, 117, 12590–12600. 10.1021/jp408166m. 24147965

[ref77] Mandal N.; Pal A. K.; Gain P.; Zohaib A.; Datta A. Transition-State-like Planar Structures for Amine Inversion with Ultralong C–C Bonds in Diamino- o -Carborane and Diamino- o -Dodecahedron. J. Am. Chem. Soc. 2020, 142, 5331–5337. 10.1021/jacs.0c00181. 32090580

[ref78] Ochi J.; Tanaka K.; Chujo Y. Recent Progress in the Development of Solid-State Luminescent o -Carboranes with Stimuli Responsivity. Angew. Chem. 2020, 132, 9925–9939. 10.1002/anie.201916666. 32009291

[ref79] Wu X.; Guo J.; Jia W.; Zhao J.; Jia D.; Shan H. Highly-Efficient Solid-State Emission of Tethered Anthracene-o-Carborane Dyads and Their Visco- and Thermo-Chromic Luminescence Properties. Dyes Pigm. 2019, 162, 855–862. 10.1016/j.dyepig.2018.11.018.

[ref80] Tahaoğlu D.; Alkan F.; Durandurdu M. Theoretical Investigation of Substituent Effects on the Relative Stabilities and Electronic Structure of [B_n_X_n_]^2–^ Clusters. J. Mol. Model. 2021, 27, 365 10.1007/s00894-021-04980-1. 34845522

