# Supplementary material for: Revisiting the Role of Charge Transfer in the Emission Properties of Carborane–Fluorophore Systems: A TDDFT Investigation
Source: J Phys Chem A. 2022 Jun 5;126(26):4199–210. doi: 10.1021/acs.jpca.2c02435 (PMC9272399; doi:10.1021/acs.jpca.2c02435)
Supplement: Supplementary file 1 — jp2c02435_si_001.pdf [file jp2c02435_si_001.pdf]

SUPPORTING INFORMATION

for

Revisiting the Role of Charge Transfer on the Emission Properties of Carborane-Fluorophore Systems: a TDDFT Investigation

Duygu Tahaoğlu,[1] Hakan Usta[1], Fahri Alkan,*[1]

[1] Department of Nanotechnology Engineering, Abdullah Gül University, Kayseri, Turkey

*Corresponding Author

E-mail: fahri.alkan@agu.edu.tr

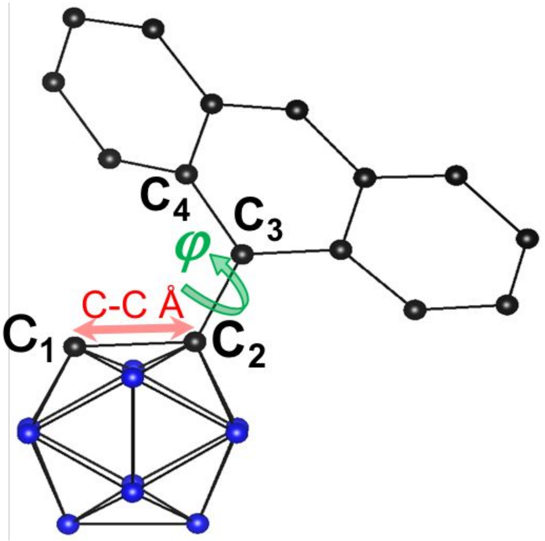

Figure S1. The illustration of important geometric parameters for excited-state geometries of *o*-CB-*Ant* along with numbering of C atoms used in the text.

Table S1. Benchmark results for the emission and absorption energies in eV (emission wavelengths in parenthesis) for different XC-functionals from the three different conformers in comparison with the experimental results.

S_1 state	Emission Energies (eV)				
	Experiment[1]	BP86	B3LYP	CAM-B3LYP	M06-2X
LE	2.76 (450)	2.20 (564)	2.39 (519)	2.59 (479)	2.61 (476)
HLCT	2.07 (600)	1.74 (713)	1.94 (639)	2.18 (570)	2.25 (551)
CT	NA	0.33 (3781)	0.74 (1670)	1.40 (887)	1.31 (947)
	Absorption Energies (eV)				
	Experiment[1]	BP86	B3LYP	CAM-B3LYP	M06-2X
LE	3.10 (400)	2.62 (474)	2.95 (420)	3.35 (370)	3.35 (370)

Table S1. Pictorial frontier orbitals of the three conformations for S_1 state along with the orbital contributions from *o*-CB and *Ant* moieties calculated with different XC functionals.

S_1 state		BP86	B3LYP	CAM-B3LYP	M06-2X
LE	LUMO	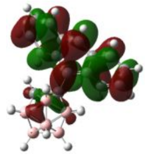 CB: 8%, Ant: 92%	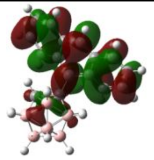 CB: 8%, Ant: 92%	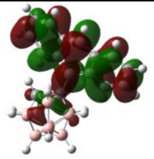 CB: 7%, Ant: 93%	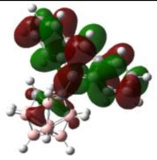 CB: 7%, Ant: 93%
	HOMO	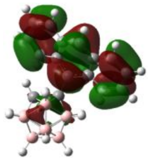 CB: 6%, Ant: 92%	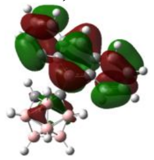 CB: 6%, Ant: 94%	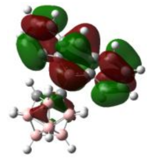 CB: 6%, Ant: 94%	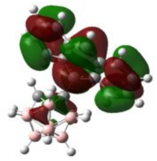 CB: 6%, Ant: 94%
HLCT	LUMO	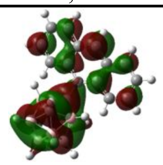 CB: 62%, Ant: 38%	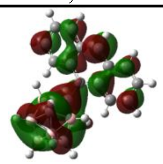 CB: 60%, Ant: 40%	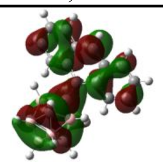 CB: 48%, Ant: 52%	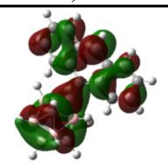 CB: 43%, Ant: 57%
	HOMO	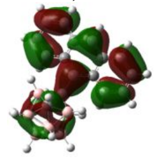 CB: 13%, Ant: 87%	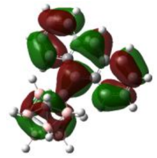 CB: 14%, Ant: 86%	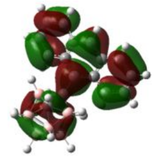 CB: 15%, Ant: 85%	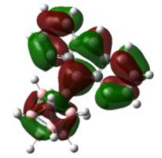 CB: 13%, Ant: 87%
CT	LUMO	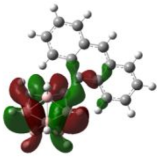 CB: 96%, Ant: 4%	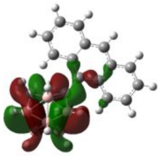 CB: 96%, Ant: 4%	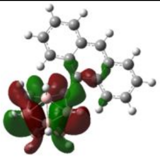 CB: 96%, Ant: 4%	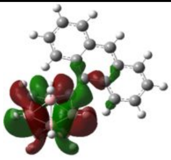 CB: 96%, Ant: 4%
	HOMO	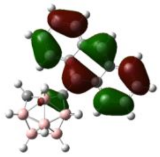 CB: 5%, Ant: 95%	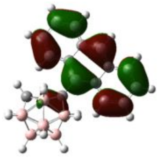 CB: 5%, Ant: 95%	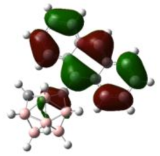 CB: 6%, Ant: 94%	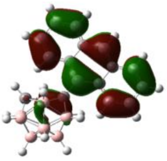 CB: 7%, Ant: 93%

Table S2. Δr and Λ values for the excited states of *o*-CB-*Ant* system with different XC functionals.

	BP86		B3LYP		CAM-B3LYP		M06-2X	
	Δr (Å)	Λ	Δr (Å)	Λ	Δr (Å)	Λ	Δr (Å)	Λ
LE	0.28	0.83	0.23	0.84	0.21	0.84	0.20	0.84
HLCT	1.78	0.65	1.68	0.67	1.06	0.71	0.92	0.73
CT	4.18	0.22	4.14	0.22	3.89	0.24	3.85	0.25

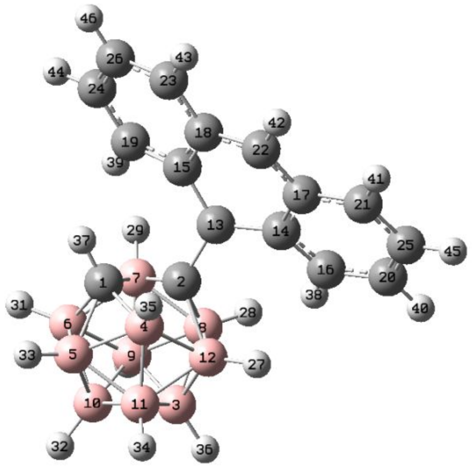

Figure S2. Atomic labels for the heat maps of TDM (H atoms are not given in the heat maps). *o*-CB atoms are numbered as 1-12 (left and down in TDM plots) whereas *Ant* atoms are numbered as 13-26. (right and up in TDM plots)

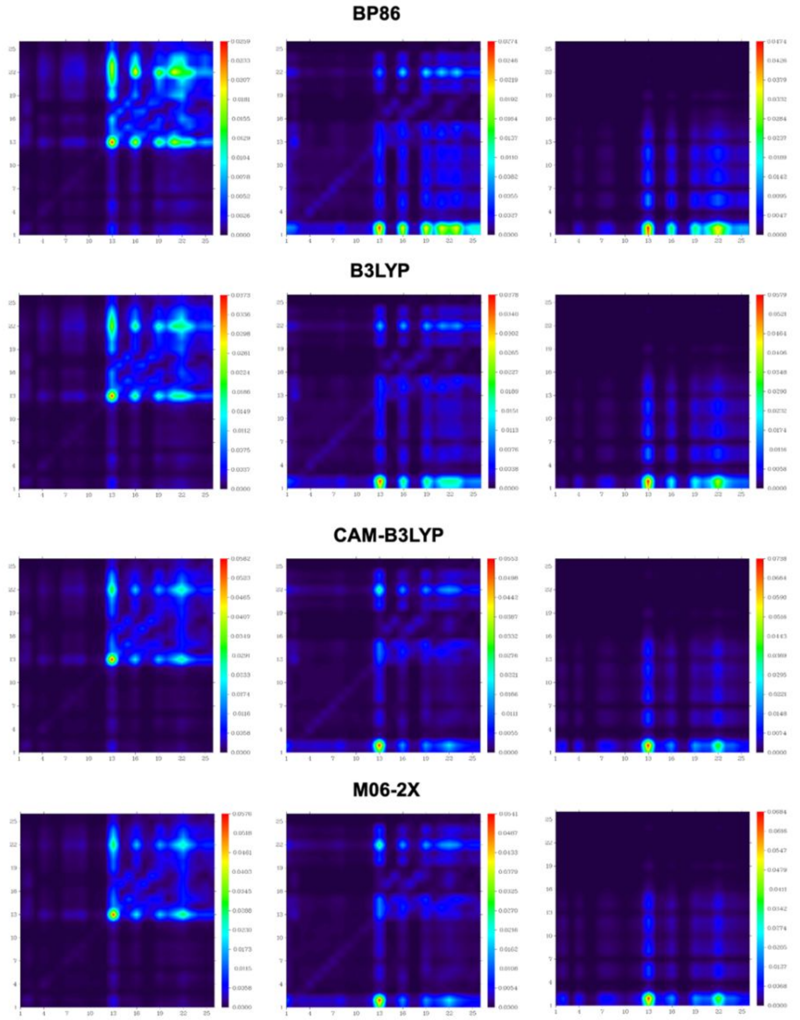

Figure S3. Heat maps of transition density matrix (TDM) graphs for S_1 states of a) LE b) HLCT and c) CT states calculated with different XC functionals.

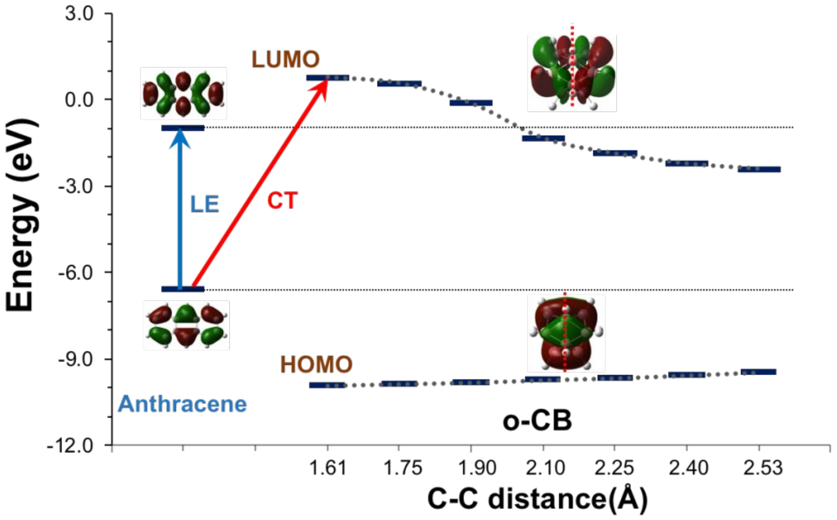

Figure S4. Relative energies of the frontier levels for *o*-CB and *Ant* moieties with respect to C_1-C_2 distance on *o*-CB.

Table S4. Δr and Λ values for the TDMs given in Figure 2 for selective $S_1 \rightarrow S_0$ transitions on the PESs with respect to φ at fixed C_1-C_2 bond lengths.

	C_1-C_2 = 1.60Å		C_1-C_2 = 2.25Å		C_1-C_2 = 2.53Å	
	Δr (Å)	Λ	Δr (Å)	Λ	Δr (Å)	Λ
φ = 0°	0.2	0.83	3.7	0.28	3.9	0.25
φ = -45°	0.2	0.82	0.8	0.75	3.1	0.47
φ = -90°	0.2	0.83	0.9	0.73	1.4	0.69

Table S5. Δr and Λ values for the TDMs given in Figure 3 for selective $S_1 \rightarrow S_0$ transitions on the PESs with respect to C_1-C_2 bond lengths at fixed φ.

	φ = -13°		φ = -87°		φ = 0°	
	Δr (Å)	Λ	Δr (Å)	Λ	Δr (Å)	Λ
C_1-C_2 = 1.60Å	0.2	0.84	0.2	0.83	0.2	0.83
C_1-C_2 = 2.00Å	0.4	0.81	0.4	0.80	0.3	0.82
C_1-C_2 = 2.50Å	3.8	0.31	1.4	0.69	3.9	0.25

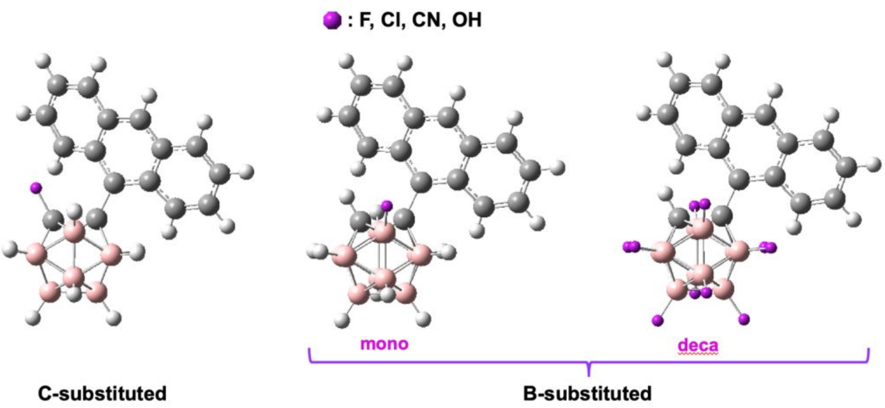

Figure S5. Positions of substituents for *o*-CB-*Ant* derivatives (X: F, Cl, CN and OH).

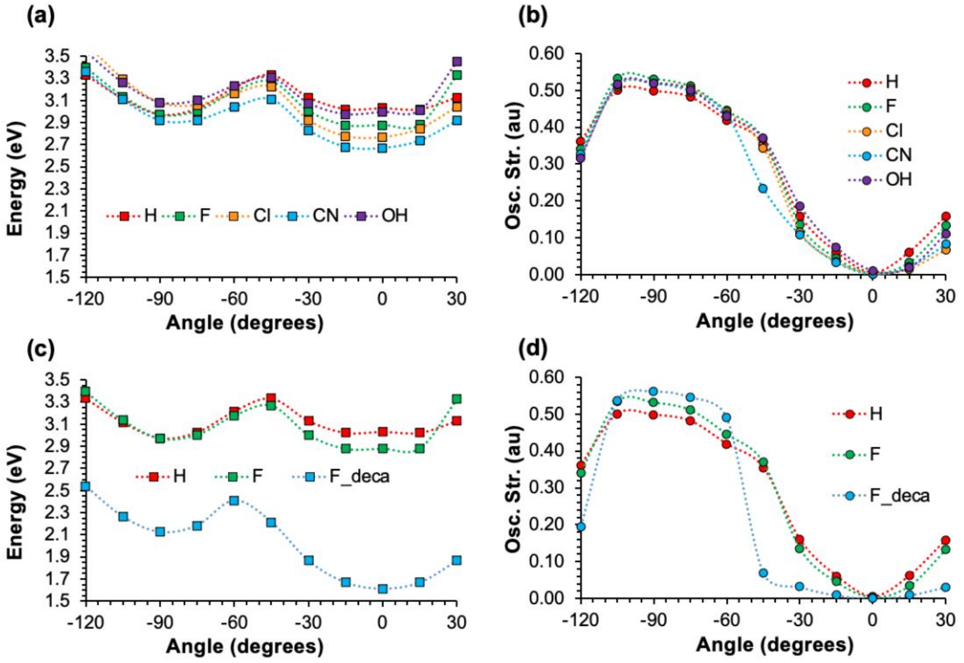

Figure S6. Potential energy surfaces with respect to φ for partially stretched and fixed C_1-C_2 bond lengths for the B-substituted derivatives of the *o*-CB-*Ant*. For these surfaces, φ = -90° corresponds to the HLCT state for each system.

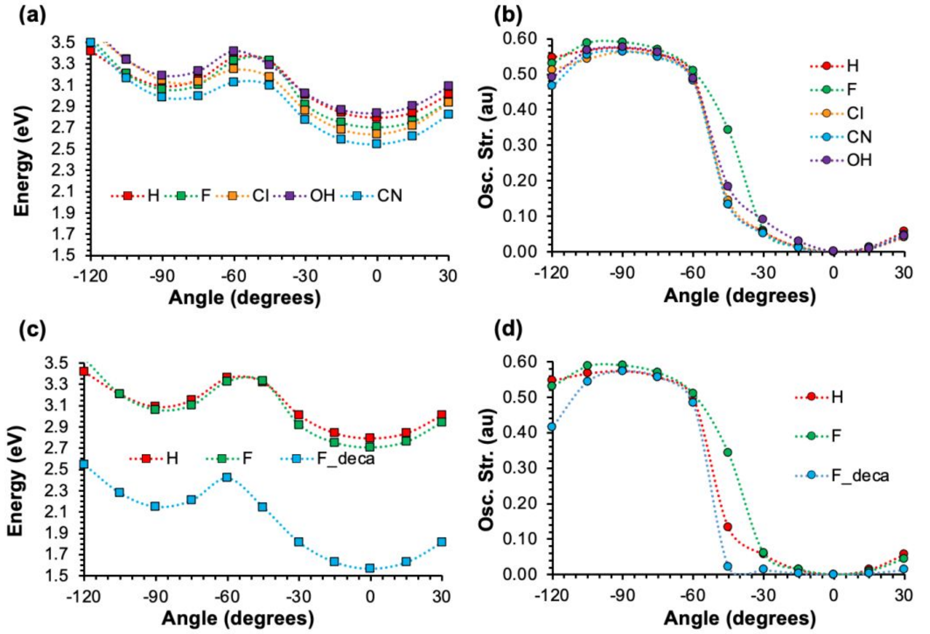

Figure S7. Potential energy surfaces with respect to φ for fully stretched and fixed C_1-C_2 bond lengths for the B-substituted derivatives of the *o*-CB-*Ant*. For these surfaces, $\varphi = 0°$ corresponds to the CT state for each system.

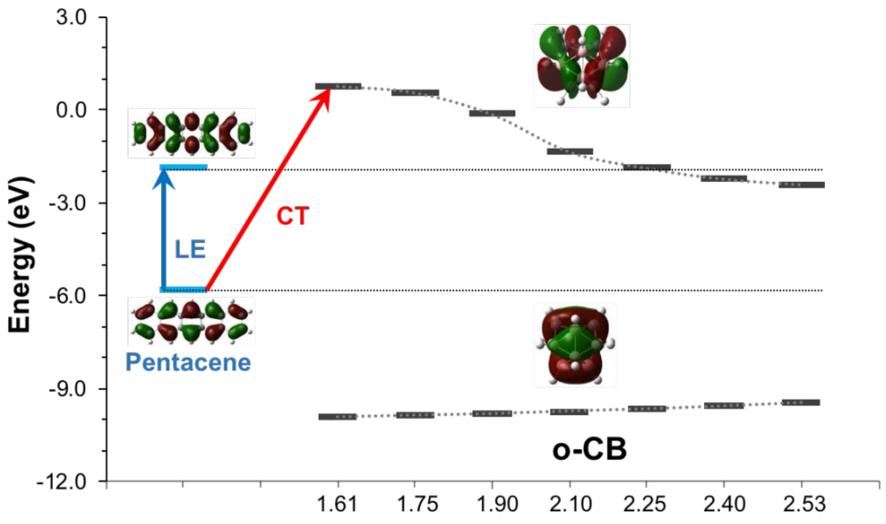

Figure S8. Relative energies of the frontier levels for *o*-CB and *Pnt* moieties with respect to C_1-C_2 distance on *o*-CB.

References

(1) Naito, H.; Nishino, K.; Morisaki, Y.; Tanaka, K.; Chujo, Y. Solid-State Emission of the Anthracene-o-Carborane Dyad from the Twisted-Intramolecular Charge Transfer in the Crystalline State. *Angew Chem Int Edit* **2017,** *56* (1), 254.
